# Imagery Rehearsal Therapy (IRT) is associated with reduced nightmare severity and depressive, anxiety and suicidal symptoms in adults with Major Depressive Episode

**DOI:** 10.1016/j.ijchp.2025.100658

**Published:** 2025-12-15

**Authors:** Julia MARUANI, Nathan MARTINS, Emmanuelle CLERICI, Michel LEJOYEUX, Pierre A. GEOFFROY

**Affiliations:** a Département de psychiatrie et d'addictologie, AP-HP, GHU Paris Nord, DMU Neurosciences, Hôpital Bichat - Claude Bernard, F-75018 Paris, France; b Université Paris Cité, NeuroDiderot, Inserm, FHU I2-D2, F-75019 Paris, France; c Centre ChronoS, GHU Paris - Psychiatrie & Neurosciences, 1 rue Cabanis, 75014 Paris, France

**Keywords:** Sleep disorders, Parasomnias, Psychotherapy, Major depressive episode, Suicidal Ideation, Controlled study

## Abstract

Introduction: Nightmare Disorder characterized by recurrent dysphoric dreams is strongly associated with major depressive episodes (MDE) and suicidal risk. Imagery rehearsal therapy (IRT) is the standard treatment for nightmares, but its effectiveness in individuals with MDE remain understudied. This study evaluated whether IRT is associated with greater improvement in nightmare symptoms compared to sleep education therapy (SET) in patients with MDE. Methods: In this non-randomized controlled study, 53 adults diagnosed with both MDE and Nightmare Disorder (DSM-5-TR criteria) received either four weekly group-based IRT sessions (*n* = 28) or a single SET session during the baseline interview, followed by a four-week waitlist before IRT (*n* = 25). Outcomes included the Nightmare Severity Index (NSI), depressive symptoms (QIDS-SR16 and HAD-D), anxiety symptoms (HAD-A and GAD-7) and suicidal ideation (item 12 of the QIDS-SR16). Treatment effects were assessed through change scores and pre- and post-intervention comparisons. Results: IRT was significantly associated with greater reductions in nightmare severity (*p* < 0.001) with improvements observed across NSI subscales: frequency (*p* = 0.010), emotional (*p* = 0.003), diurnal (*p* = 0.017), and nocturnal impacts (*p* = 0.002). Associations were also found between IRT and reductions in depressive (ΔHAD-D *p* < 0.001, ΔQIDS-SR16 *p* = 0.028), anxiety (ΔHAD-A and ΔGAD-7 *p* < 0.001) and suicidal symptoms (*p* = 0.002). Treatment-resistant depression predicted greater improvements in the emotional impact of nightmares (*p* = 0.007), while nightmare frequency was associated with reduced benefit (*p* = 0.008). Conclusion: IRT is associated with meaningful reductions in nightmare severity, depressive symptoms, anxiety, and suicidal ideation in individuals with MDE. These findings support the integration of IRT in treatment plans for this high-risk population.

## Introduction

Chronic nightmares, also known as Nightmare Disorder, are severe condition defined by the third edition of the International Classification of Sleep Disorders (Sateia, 2014) and the Diagnostic and Statistical Manual of Mental Disorders, Fifth Edition, Text Revision (Diagnostic & Statistical Manual of Mental Disorders, s. d., 2023) as repeated and lengthy dreams, extremely dysphoric, with clear memory, typically involving life-threatening threats to safety or physical integrity (Sateia, 2014). These nightmares lead to significant distress upon awakening, although the individual quickly regains alertness. The experiencing of these dreams, or the disruption of sleep produced by the ensuing awakening, causes significant distress in daily life or impairment in the person's functioning. Nightmare Disorder is overrepresented in psychiatric disorders. It is estimated to affect 2 to 8 % of the general population, compared to over a third among young individuals entering psychiatric care (Akkaoui et al., 2022). In patients with Major Depressive Episode (MDE), the prevalence increases to 16.7 % and rise to up to 90 % when associated with melancholic features or suicidal behaviours (Akkaoui et al., 2020). In these patients, nightmare themes often revolve around despair, loss, or death, which further reinforce daytime depressive symptoms. Moreover, nightmares are associated with poor sleep quality, early morning awakenings, and increased fatigue and depressive mood (Sjöström et al., 2009). The emotional impact of nightmares seems to be more decisive than their frequency. Indeed, it is the negative emotional charge of dreams that correlates with the severity of depression rather than the simple number of episodes. In addition, nightmares often cause night time awakenings and insomnia, further exacerbating the symptoms of despair and fatigue characteristic of depression (Akkaoui et al., 2022). Importantly, sleep complaints have been found to predict suicidal behaviours independently of any underlying psychopathology (Geoffroy et al., 2021). Among these sleep disturbances, nightmares seem to be one of the most predictive factors for these behaviours during depression (Geoffroy et al., 2022). It has been observed that over 80 % of individuals experiencing a suicidal crisis (including suicide attempts or suicidal ideation with immediate intent) alter their dream content in the months preceding this crisis (Geoffroy et al., 2022). A progression of dream content alteration seems to be observed, with bad dreams (dysphoric dreams) appearing approximately 4 months before the crisis, nightmares (bad dreams that awaken the sleeper) occurring 3 months before, and suicidal scenarios in dreams emerging around 1.5 months before the onset of the crisis (Geoffroy et al., 2022). Nightmares may also predict suicide recurrence. A prospective study involving 165 patients hospitalized for a suicide attempt found that those reporting frequent nightmares were three times more likely to attempt suicide again within two years (Geoffroy et al., 2022). This risk increased further if the nightmares persisted two months into follow-up (Sjöström et al., 2009). In patients with persistent depression and frequent nightmares, recurrence rates reached 87 %. While the exact mechanisms behind these associations remain unclear, several hypotheses have been proposed. Nightmares may increase emotional distress, worsen sleep disturbances, and amplify negative thinking and suicidal ruminations (Akkaoui et al., 2022; Geoffroy et al., 2022). They can be understood as nocturnal manifestations of daytime psychological distress, particularly among individuals with mood disorders (Akkaoui et al., 2022; Schredl, 2016). These findings underlie the urgent need to treat patients with a major depressive episode and co-occurring nightmare disorder to prevent the progression of depressive symptoms into a possible suicidal crisis, particularly addressing the nightmare disorder. Imagery Rehearsal Therapy (IRT) stands as the first-line treatment for Nightmare Disorder in international therapeutic recommendations (Aurora et al., 2010; Krakow & Zadra, 2010; Krakow et al., 1995; Morgenthaler et al., 2018; Romier et al., 2024). Built upon the principle of Continuity Theory (Domhoff, 2001), IRT posits a seamless connection between waking life and dream states, allowing the brain to integrate daytime experiences into nocturnal narratives. Moreover, it perceives nightmares as modifiable behaviours (Krakow et al., 1995), enabling cognitive and behavioural restructuring through the exploration of nightmare scenarios with positive mental imagery (Romier et al., 2024). IRT is widely recognized for its effectiveness in treating idiopathic chronic nightmares or those associated with Post-Traumatic Stress Disorder (PTSD) with no reported adverse effects. Studies have demonstrated a reduction in nightmare frequency and associated distress for up to 3 months with maintained efficacy for as long as 30 months (Augedal et al., 2013; Hansen et al., 2013; Krakow et al., 1993). Additionally, IRT has shown efficacy in a diverse psychiatric population (van Schagen et al., 2015). Interestingly, regarding major depressive episode, a first open-label and non-controlled study, demonstrated the effectiveness of IRT in reducing nightmare frequency, decreasing anxiety symptoms, depressive symptoms, next-day distress, and nocturnal awakenings in patients experiencing depression with nightmares (Thünker & Pietrowsky, 2012). Additionally, a second open-label and non-controlled study illustrated the effectiveness of IRT in reducing suicidal ideation, showing a significant effect size in Major Depressive Episode with nightmares (Ellis et al., 2019). However, there is a lack of evidence with control study on the efficacy of IRT in patients with MDE and Nightmare Disorder. Furthermore, no study has been undertaken to assess the efficacy oof IRT on nightmares in depression using the Nightmare Severity Index (NSI). The NSI is especially interesting since this is a global multidimensional scale that encompasses all primary dimensions of nightmare disorder as defined by the DSM-5-TR and ICSD-3, including the frequency, emotional impact, diurnal impact, and nocturnal impact of nightmares (Geoffroy et al., 2024). In this study, conducted as part of routine clinical care and involving patients with major depressive episodes (MDE), the aim was to compare an IRT treatment group with a control group that received Sleep Education Therapy (SET) during the baseline interview while awaiting IRT treatment for nightmare disorder. The outcomes included the severity of nightmare disorder, assessed using the Nightmare Severity Index (NSI), as well as depressive symptoms and suicidality in patients with MDE.

## Methods

The primary objective of this study was to explore whether IRT was associated with improvements in nightmares severity among patients with a major depressive episode, performed into routine care in the Department of Psychiatry, AP-HP, and at the Chronos Center (Psychiatry, Chronobiology, and Sleep) of the Paris Psychiatry Neuroscience University Hospital Group. The second aim of this study was to evaluate whether IRT was also associated with reductions in depressive symptoms and suicidal ideation and to identify potential predictive factors of treatment response. Patients aged between 18 and 70 years were diagnosed with Nightmare Disorder and MDE by a psychiatrist and sleep physician according to the Structured Clinical Interview for DSM-5-TR criteria (American Psychiatric Association, 2013). Patients who did not exhibit clinical symptoms of depression according to the DSM-5-TR diagnostic criteria were not considered. Patients could have pharmacological treatment, which was not modified during the study period. Routine treatment consisted of 4 (120-min) sessions of group IRT delivered weekly in a structured sequence according to the standardized protocol developed by the team (Stern et al., 2022). IRT consists of 4 sessions: I) therapeutic education and pathophysiology of nightmares, II) practice of mental imagery, III) application of mental imagery techniques to nightmares with transcription of the scenario and participant training in IRT at home, and IV) relapse prevention session and assessment. The core of IRT involves reworking the nightmare scenario using mental imagery and practicing exercises several times a day between sessions. The control group was a waiting list made of patients waiting for 4 weeks to start the IRT treatment and that received sleep education therapy at baseline interview (SET). SET targets day-to-day behavioural and environmental factors that contribute to poor sleep and was performed for the control group during a single session (Morin et al., 2006). The four IRT sessions and the SET session were delivered by the same trained psychologist specialized in IRT, along with a trained medical doctor. In the IRT group, participants were asked to engage starting from session 2, in daily mental imagery exercises individually at home. In addition, they were asked to complete a daily dream log, in which they recorded the occurrence and frequency of nightmares, the associated emotional valence and intensity, as well as any adverse reactions. We compared two groups: the intervention group and the waiting list who benefited from SET. The evolution of the characteristics of the control group was evaluated using sleep, psychiatric and addictive questionnaires at baseline and pre-IRT, (immediately prior to entering the IRT program), while the evolution of the characteristics of the IRT group was assessed using pre-IRT (i.e., before the first IRT session) and post-IRT measurements, a few weeks after the end of the IRT group. The primary outcome was the change in nightmare symptom severity from baseline to post-treatment, assessed using the Nightmare Severity Index (NSI) (Geoffroy et al., 2024) in order to evaluate the association between IRT and improvements in nightmare-related symptoms. Secondary criteria included improvement in each sub score of the NSI from baseline to post-treatment, as well as response to IRT, defined as a reduction of more than 50 % in the severity score of the NSI, along with responses 25 % and 33 %. Our secondary aim was to assess the impact of IRT on depressive symptoms using the Hospital Anxiety and Depression Scale (HAD) (Snaith, 2003), and its subscale HAD-D and HAD-A. Additionally, we examined changes with the IRT on suicidal ideation using a single item (item 12) extracted from the French Quick Inventory of Depressive Symptomatology Self-Report 16 items (QIDS-SR16) questionnaire, which is also validated for depression assessment (Deneuve, s. d.; Rush et al., 2003). The item specifically addressed ideation of death or suicide (0: I do not think about suicide or death; 1:1 I think life is meaningless or I wonder if it's worth living; 2: I think about suicide or death several times a week for several minutes; 3: I think about suicide or death several times a day in detail, I have considered suicide specifically, or I have actually attempted suicide). Suicidal ideation response to IRT was also compared between the two groups (reduction of more than 50 % of patients with suicidal ideation) along with 25 % and 33 % responses. Finally, we conducted a linear regression analysis predicting posttreatment nightmares improvement and logistic regression analysis predicting posttreatment nightmare response. Predictive factors were sought from sociodemographic characteristics, personal and family background, mood-related characteristics, sleep, and nightmares obtained during the initial interview.

All patients received care at Bichat Hospital, Department of Psychiatry, AP-HP, and at the Chronos Center (Psychiatry, Chronobiology, and Sleep) of the Paris Psychiatry Neuroscience University Hospital Group. They participate in IRT groups for nightmare treatment. This project is part of the "Som-Psy" routine care cohort: Study of sleep and circadian rhythms biomarkers in psychiatric disorders promoted by Assistance Publique - Hôpitaux de Paris with the favorable opinion (No. CER-2020–56) of the Committee for Evaluation of the Ethics of Biomedical Research Projects (CEERB) Paris Nord (Institutional Review Board - IRB 00,006,477- of the HUPNVS, Paris 7 University, AP-HP). Patients have received a non-opposition information note regarding the purpose of this study and that clinical information may be used for research.

### Sleep and circadian rhythms measures

Patients completed the French versions of sleep and circadian rhythms questionnaires. To assess nightmare severity, we used the Nightmare Severity Index (NSI) (Geoffroy et al., 2024), a validated, multidimensional tool aligned with DSM-5-TR and ICSD-3 criteria for Nightmare Disorder. The NSI was chosen because it captures the four key dimensions of nightmare pathology—frequency, emotional impact, diurnal impact, and nocturnal impact—providing a comprehensive and clinically relevant profile of nightmare severity. The NSI proposes a total score ranging from 0 to 20 and is derived from nine items that yield sub-scores across the four dimensions (Geoffroy et al., 2024). Higher scores indicate greater nightmare severity. English and French versions of the NSI are freely available to download and use. The total NSI score is calculated by adding up the scores from four subscales, each representing a distinct dimension of nightmares. Each sub score ranges from 0 to 5 and captures the following aspects: nightmare frequency (NSI SS1), emotional impact (NSI SS2), diurnal impact (NSI SS3), and nocturnal impact (NSI SS4), as assessed through specific questions. Consequently, the total score is obtained by adding the scores from the four subscales: NSI SS1 for nightmare frequency is based on responses to Q1- Please estimate how often your bad dreams wake you up -, NSI SS2 for emotional impact is determined by the highest score between responses to Q2- Please estimate your nightmares ‘emotional intensity- and Q3- Please estimate how often your nightmares involve vital threats to safety or physical integrity, NSI SS3 for diurnal impact is calculated as the highest score between responses to Q4 to Q7 (Q4Please estimate how your nightmares interfere with your global functioning Diurnal impact, Q5 Please estimate how your nightmares interfere with your mood during the day Diurnal impact, Q6 Please estimate how your nightmares interfere with your concentration/memory during the day, Diurnal impact Q7 Please estimate how your nightmares disturb your arousal ability (sleepiness) during the day), and NSI SS4 for nocturnal impact is derived from the highest score between responses to Q8- Please estimate how your nightmares interfere with falling asleep or Q9- Please estimate how your nightmares interfere with your sleep continuity.

Insomnia symptoms in the past month were assessed with the Insomnia Severity Index (ISI) (Bastien et al., 2001), validated in French, in a population with psychiatric disorders and sleep complaints (Stern et al., 2025). It includes seven items, yielding a total score ranging from 0 to 28. Higher scores indicate more severe insomnia symptoms A score of 8 or higher is considered the clinical threshold for significant insomnia symptoms. All participants were also assessed regarding their sleep quality during the past month with the French version of the 19-item Pittsburgh Sleep Quality Index (PSQI) (Ait-Aoudia et al., 2013), using the total score and the seven PSQI sub scores: subjective sleep quality, sleep latency, sleep duration, habitual sleep efficiency, sleep disturbance, use of night sedation, and daytime dysfunction. A total score ≥5 indicates a significantly disturbed sleep, and rating of the seven components indicates altered dimensions with increasing sub scores. Participants also completed the Epworth Sleepiness Scale (ESS) (Johns, 1991), an instantaneous subjective measure of daytime sleepiness (Johns, 1991). A total score of ≥11 on the ESS indicates pathological sleepiness. Finally, participants completed a chronotype scale the Horne & Östberg questionnaire (Horne & Ostberg, 1976) selected for its validated ability to characterize circadian rhythm preference, which has been shown to influence mood, sleep patterns, and treatment outcomes in psychiatric populations.

### Sociodemographic and clinical parameters

Sample sociodemographic characteristics such as age and gender were collected. Other clinical parameters were assessed including the age of onset, the type of mood disorder (unipolar or bipolar). To comprehensively assess mood symptoms and suicidal risk in patients with MDE and Nightmare Disorder, we selected the Hospital Anxiety and Depression Scale (HADS), the Generalized Anxiety Disorder-7 (GAD-7), and the Quick Inventory of Depressive Symptomatology – Self-Report (QIDS-SR16) based on their complementary strengths, clinical relevance, and robust validation in psychiatric populations. The HADS (Snaith, 2003), and its subscale HAD-D and HAD-A were specifically designed to evaluate depression and anxiety symptoms in medical and psychiatric populations, while excluding symptoms such as fatigue or sleep disturbance which may overlap with others medical conditions. Depressive symptoms were further assessed with the Quick Inventory of Depressive Symptomatology – Self-Report (QIDS-SR16), which is validated in French and captures all DSM-5-TR depressive domains (e.g., mood, energy, sleep, appetite, psychomotor symptoms). This tool is widely used in both clinical trials and real-world settings. Item 12 of the QIDS-SR16 specifically evaluates suicidal thoughts and behaviors. Other questionnaires were used to assess psychiatric symptoms, such as the Generalized Anxiety Disorder-7 (GAD-7) (Spitzer et al., 2006) to provide a more specific assessment of generalized anxiety severity for the past 15 days. Furthermore, the characteristics of depression were assessed, including treatment-resistant depression (defined as the failure of at least two adequate antidepressant trials (in terms of dose and duration, based on treatment history and clinical records, in accordance with standard criteria) and we investigated psychiatric and addictive comorbidities.

### Statistical analysis

As an initial step, we compared all sample characteristics to determine whether the two groups were equivalent (see Table 1). For variables that differed between groups, we conducted a multivariate analysis (see Table 4) to assess whether the observed effect of IRT could be attributed to these baseline differences. Subsequently, we assessed the impact of IRT on nightmares by comparing the SET and IRT groups (see Table 2). Following this, we examined the influence of IRT on depressive symptoms and suicidal ideation between the two groups (see to Table 3). Quantitative results were expressed as means and standard deviations, and categorical variables as percentages. The normality of the continuous variables’ distribution was assessed with visual inspection of histograms and with the statistical test of normality (Shapiro-Wilk test *p* > 0.05), and we looked at the equality of variances between the 2 groups for the measured variable (Levene test *p* > 0.05). We compared groups using parametric or non-parametric tests in case of non-normal distribution. In addition, as a sensitivity analysis, we conducted linear mixed-effects models to examine group × time interaction effects to test the robustness of our main findings. Given the small sample size (*N* = 53), these models were considered exploratory.

**Table 1:** Sample sociodemographic and clinical characteristics of patients with nightmare and major depressive disorder treated with Imagery Rehearsal Therapy (IRT) and Sleep Education Therapy (SET) (NSI=Nightmare severity index; ISI=Insomnia severity index; ESS=Epworth sleepiness scale; PSQI=Pittsburgh sleep quality index; GAD-7= Generalized Anxiety Disorder-7; HAD=Hospital anxiety and depression scale; QIDS-SR16=Quick inventory depressive symptoms-self report 16 items; SUD=Substance use disorder; MDE=Major depressive disorder; TRD=Treatment resistant-depression).

	IRT (n =28)	SET (n=25)	P-value	T or U or X2
	*mean (SD) or N (%)*	*mean (SD) or N (%)*		
**Gender***Male*	9(32,1%)	8(32%)	0,991	X2
*Female*	19(67,9%)	17(68%)		
**Age**, *mean (sd)*	38,64(15,00)	42,64(16,03)	0,412	U
*Sleep variables*				
**Horne and Ostberg**	46,21(8,1)	45,75(8,6)	0,842	T
**NSI – Nightmares Severity**	14,92(3,643)	13,95(3,979)	0,398	T
**NSI SS1 Frequency**	3,35-1,441)	3,1(1,334)	0,472	U
**NSI SS2 Emotional Impact**	4,23(0,908)	4,05(1,079)	0,630	U
**NSI SS3 Diurnal Impact**	3,58(1,270)	3,32(1,336)	0,507	U
**NSI SS4 Nocturnal Impact**	3,77(1,243)	3,32(1,529)	0,335	U
**ISI Total Score**	15,87(4,875)	18,24(4,612	0,078	T
**ISI1a - Severity of sleep latency**	1,63(1,149)	2(1,216)	0,269	T
**ISI1b - Difficulty staying asleep**	2,07(1,207)	2,60(1,118)	0,137	U
**ISI1c - Early morning awakening**	1,7(1,409)	2,21(1,56)	0,240	U
**ISI2 - Satisfaction with current sleep**	3,04(0,94)	3,36(0,638)	0,227	U
**ISI3 - Disruption of daily functioning**	3,04(0,94)	2,96(0,978)	0,505	U
**ISI4 - Apparent difficulties**	1,94(1,243)	2,36(1,221)	0,255	U
**ISI5 - Concerns regarding sleep disturbances**	2,72(0,859)	2,92(0,862)	0,334	U
**ESS**	11,22(4,933)	9,29(5,996)	0,213	T
**PSQI**	11,48(3,355)	12,35(3,613)	0,384	T
**PSQI Sleep subjective quality**	2,15(0,770)	2,43(0,507)	0,211	U
**PSQI Sleep latency**	**1,44(1,05)**	**2,04(1,022)**	**0,039**	U
**PSQI Sleep duration**	1,19(1,331)	1(1,206)	0,695	U
**PSQI Sleep efficiency**	1,44(1,251)	1,26(1,251)	0,619	U
**PSQI Sleep disturbance**	1,96(0,587)	1,96(0,706)	0,965	U
**PSQI Sleep promoting medication**	1,69(1,408)	2,04(1,331)	0,323	U
**PSQI Sleep Dysfunction**	1,8(0,866)	1,61(0,656)	0,404	U
*Severity of Depression and anxiety*				
**QIDS-SR16**	12,61(5,45)	13,043(4,63)	0,762	T
**HAD**	21,59(6,85)	20,76(6,83)	0,663	T
**HAD-D**	8,63(4,12)	8,64(4,27)	0,993	T
**HAD-A**	12,96(4,2)	12,12(4,15)	0,320	U
**GAD-7**	11,68(6,01)	12,043(4,92)	0,816	T
**Suicidal Ideation**	1,18(1,44)	0,783(1,2)	0,335	U
**Suicidal Ideation***Yes*	15(53,6%)	10(43,5%)	0,473	X2
*No*	13(46,4%)	13(56,5%)		
*Comorbidities*				
**Anxiety disorder***Yes*	6(21,4%)	10(40%)	0,142	X2
*No*	22(78,6%)	15(60%)		
**SUD***Yes*	4(17,4%)	5(26,3%)	0,483	X2
*No*	19(82,6%)	14(73,7%)		
**MDE with TRD***Yes*	3(14,3%)	1(5,6%)	0,370	X2
*No*	18(85,7%)	17(94,4%)		
**MDE with anxiety***Yes*	12(63,2%)	10(55,6%)	0,638	X2
*No*	7(36,8%)	8(44,4%)		
**Current alcohol use disorder***Yes*	2(8,7%)	3(15,8%)	0,480	X2
*No*	21(91,3%)	16(84,2%)		
**Current cannabis use***Yes*	1(4,3%)	1(5,3%)	0,890	X2
*No*	2295,7%)	18(94,7%)		
**Current tobacco smoking***Yes*	8(33,3%)	9(39,1%)	0,679	X2
*No*	16(66,7%)	14(60,9%)		
**Personality disorder***Yes*	4(16,7%)	3(15%)	0,880	X2
*No*	20(83,3%)	17(85%)		
**Trauma stress disorder***Yes*	5(17,9%)	4(16%)	0,857	X2
*No*	23(82,1%)	21(84%)		
**Treatments**				
**Antidepressants***Yes*	13(81,3%)	13(76,5%)	0,737	X2
*No*	3(18,8%)	4(23,5%)		
**Anxiolytics***Yes*	7(41,2%)	8(44,4%)	0,845	X2
*No*	10(58,8%)	10(55,6%)		
**Mood stabilizers***Yes*	3(18,8%)	0(0%)	0,054	X2
*No*	13(81,3%)	18(100%)		
**Hypnotics***Yes*	4(23,5%)	6(33,3)	0,521	X2
*No*	13(76,5%)	12(66,67)		
**Antipsychotics***Yes*	3(17,6%)	1(5,6%)	0,261	X2
*No*	14(82,4%)	17(94,4%)

**Table 2:** Comparison of efficacy of Imagery Rehearsal Therapy (IRT) and Sleep Education Therapy (SET) on nightmare and sleep (NSI=Nightmare severity index; ISI=Insomnia severity index; ESS=Epworth sleepiness scale; PSQI=Pittsburgh sleep quality index).

	IRT (n=28)	SET (n=25)	Test (t student or U Mann Whitney	P-value	Δ mean (Δ SD)	95% CI	Effect sizes (Cohen’s d)	Effect sizes (Hedges’ g)
	*mean (SD) or N (%)*	*mean (SD) or N (%)*						
***Sleep parameters,***								
**NSI - Nightmares Severity**	-3							
*Δ NSI Total Score*	-3,5769(3,4)	1,1579(3,76)	U	**<0,001**	4	**2,000 – 6,000**	**1,3326**	**1,3133**
*Δ NSI-SS1 (Nightmare Frequency)*	-0,96(1,248)	0,0526(1,129)	U	**0,010**	1	**1,17e-5 – 2, 000**	**0,8452**	**0,8326**
*Δ NSI-SS2 (Nightmare Emotional impact)*	-1,04(1,148)	0(0,943)	U	**0,003**	1	**6,87e-5 – 1,000**	**0,973**	**0,9587**
*Δ NSI-SS3 (Nightmare Diurnal impact)*	-0,65(1,5)	0,4737(1,389)	U	**0,017**	1	**6,60e-5 – 2,000**	**0,777**	**0,7656**
*Δ NSI-SS4 (Nightmare Nocturnal impact)*	-0,92(1,129)	0,6316(1,495)	U	**0,002**	1	**4,90e-5 – 2,000**	**1,125**	**1,1082**
**NSI response**			X2	0,073				
*Yes*	4(15,4%)	0(0%)						
*No*	22(84,6%)	19(100%)						
**NSI 33 % response**			X2	**0,004**				
*Yes*	9(34,6%)	0(0%)						
*No*	17(65,4%)	19(100%)						
**NSI 25 % response**			X2	**<0,001**				
*Yes*	12(46,2%)	0(0%)						
*No*	14(53,8%)	19(100%)						
**ISI**								
*Δ ISI Total score*	-1,77(3,653)	-1(4,041)	T	0,488	0,7708	-1,4462 – 2, 9879	0,2	0,1970
*Δ ISI1a –***Severity of sleep latency**	-0,042(1)	-0,1667(0,702	U	0,59	-4,26e-5	-1,000 – 6,72^e^-5	-0,145	-0,1429
*Δ ISI1b –***Difficulty staying asleep**	-0,047(0,86)	-0,16(0,987)	U	0,57	0	-1, 000 -3,14e-5	-0,128	-0,1261
*Δ ISI1c –***Early morning awakening**	-0,417(0,97)	-0,417(1,176)	U	0,593	7,82e-5	-4,49^e^−5 – 1,000	0	0
*Δ ISI2 –***Satisfaction with current sleep**	-0,417(0,78)	-0,08(0,640)	U	0,062	7,13^e^-5	-5,31^e^−5–1,000	0,474	0,4669
*Δ ISI3 –***Disruption of daily functioning**	-0,229(0,91)	-0,16(0,688)	U	0,595	8,54^e^-5	-6,32e−5 –1,000	0,086	0,0847
*Δ ISI4 –***Apparent difficulties**	-0,104(0,93)	-0,16(0,898)	U	0,949	8,94e-5	- 1,000 -1,000	-0,06	0,0591
*Δ ISI5 –***Concerns regarding sleep disturbances**	-0,521(0,74)	0(0,577)	U	**0,005**	4,06e-6	7.82e-5-1.000	**0,7841**	**0,7726**
**PSQI**								
Δ	-1,37(3,93)	-0,043(2,38)	U	**0,038**	2	3,62e-5 – 3,000	**0,4074**	**0,4014**
*ΔPSQI Sleep subjective quality*	-0,33(0,96)	-0,17(0,388)	U	0,434	3,04e-5	-2,99e−5 – 1,000	0,2155	0,2122
*ΔPSQI sleep latency*	0,0417(0,86)	-0,478(0,79)	U	0,050	-4,78^e^-5	-0,0201 – 1,2882	-0,629	-0,6195
*ΔPSQI Sleep duration*	-0,417(1,25)	0,217(0,951)	U	**0,033**	3,86e-6	-1,85e−5 –1,000	**0,57**	**0,5616**
*ΔPSQI Sleep dysfunction*	-0,09(1,06)	0,19(0,75)	U	0,209	2,55e-6	-4,63^e^−5 – 1,000	0,304	0,2995
*ΔPSQI Sleep efficiency*	-0,29(1,27)	0,4(0,99)	U	**0,022**	1	5,24e−5 – 1,000	**0,60**	**0,5911**
*ΔPSQI Sleep disturbance*	-0,17(0,48)	-0,043(0,367)	U	0,320	3,52e-5	-4,28e−5 - 7,02e-5	0,29	0,2857
*ΔPSQI Sleep promoting medication*	-0,26(1,214)	-0,043(1,147)	U	0,299	0	-6,69e−5 – 2,12e-5	0,184	**0,1812**
**ESS**								
*ΔESS*	-1,043(3,04)	-0,625(1,663)	T	0,559	5,87e-5	-1,0120 – 1,8489	0,172	0,1695
**Horne and Ostberg**								
*Δ Horne and Ostberg*	1,87(4,82)	-0,08(2,95)	U	0,065	-2	-5,000 – 1,01e-5	-0,491	-0,4836

**Table 3:** Comparison of efficacy of Imagery Rehearsal Therapy (IRT) and Sleep Education Therapy (SET) on depression and anxiety symptoms in patients with major depressive episode (MDE) (GAD-7= Generalized Anxiety Disorder-7; HAD=Hospital anxiety and depression scale; QIDS-SR16=Quick inventory depressive symptoms-self report 16 items).

	IRT (n=28)	SET (n=25)	Test (t student / U Mann Whitney / T de Welch W	P-value	Δ mean (Δ SD)	95% CI	Effect sizes (Cohen’s d)	Effect sizes (Hedges’ g)
	*mean (SD) or N (%)*	*mean (SD) or N (%)*						
***Mood parameters, mean (SD)***								
**ΔHAD-D**	-1,815(3,1)	0,12(1,81)	U	**<0,001**	2	**1,00 – 4, 00**	**0,755**	**0,744**
**ΔHAD-A**	-3,78(3,83)	0,48(1,83)	U	**<0,001**	5	**3,00 – 6,00**	**1,402**	**1,381**
**ΔHAD**	-5,593(6,3)	0,6(2,9)	U	**<0,001**	7	**5,00 – 9,00**	**1,251**	**1,232**
**HAD response**			X2	**0,045**				
*Yes*	4(14,8%)	0(0%)						
*No*	23(85,2%)	25(100%)						
**HAD-A response**			X2	**0,012**				
*Yes*	6(22,2%)	0(0%)						
*No*	21(77,8%)	25(100%)						
**HAD-D response**			X2	**0,003**				
*Yes*	8(29,6%)	0(0%)						
*No*	19(70,4%)	25(100%)						
**HAD-D response 33%**			X2	**<0,001**				
*Yes*	12(44,4%)	1(4%)						
*No*	15(55,6%)	24(96%)						
**HAD-D response 25%**			X2	**<0,001**				
*Yes*	15(55,6%)	2(8%)						
*No*	12(44,4%)	23(92%)						
**ΔGAD-7**	-2,964(3,92)	0,652(2,71)	W	**<0,001**	3,62	**1,677 – 5,56**	**1,074**	**1,058**
**ΔSuicidal Ideation**	-0,75(1,11)	0,261(1,01)	U	**0,002**	2,31e-5	**4.84e-6 – 1.00**	**0,948**	**0,933**
**Suicidal Ideation response**			X2	**0,013**				
*Yes*	11(39,3%)	2(8,7%)						
*No*	17(60,7%)	21(91,3%)						
**ΔQIDS**	-1,815(3,1)	0,522(3,15)	T	**0,028**	2,38	**0,227 – 4,49**	**0,638**	**0,628**

Then, we conducted a linear regression analysis to identify possible predictors, of our principal aim: Nightmares, with NSI post-treatment improvement followed by a multivariate model incorporating all factors that proved significant in the initial univariate step (*p* < 0,05; refer to Table 5). We next searched predictors for all NSI sub scores, also followed by a multivariate model for NSI SS2 (refer to Table 6) and for NSI SS4 (refer to Table 7). All analyses were carried out using JAMOVI software (version 2.3). In all tests, a significance level of *p* < 0.05 was considered as indicative of statistical significance.

### Study sample and baseline characteristics

A total of 53 adult patients with MDE and Nightmare Disorder received routine care, either IRT or SET. Among them, 28 patients were assigned to the IRT group, and 25 to the control group receiving Sleep Education Therapy (SET) during a 4-week waiting period. All patients attended all scheduled sessions and completed the daily dream logs consistently. No serious adverse events occurred. Baseline sociodemographic and clinical characteristics of the two groups are summarized in Table 1. There were no significant differences between groups in age, sex, nor in clinical measures related to depressive and anxiety symptoms (HADS: *p* = 0.663; HAD-D: *p* = 0.993; HAD-A: *p* = 0.320), suicidal ideation (QIDS-SR16 item 12: *p* = 0.335), or nightmare severity (NSI total score: *p* = 0.398). Likewise, no significant differences were observed across sleep-related variables (PSQI, ISI, ESS), except for sleep latency on the PSQI (*p* = 0.039), with the IRT group reporting a shorter latency at baseline. There were also no significant differences between groups in psychiatric comorbidities or medication use at inclusion. This similarity in baseline characteristics between the groups supports the comparability of the IRT and SET groups prior to intervention.

## Results

### IRT effect on nightmares and sleep

**Fig. 1:**
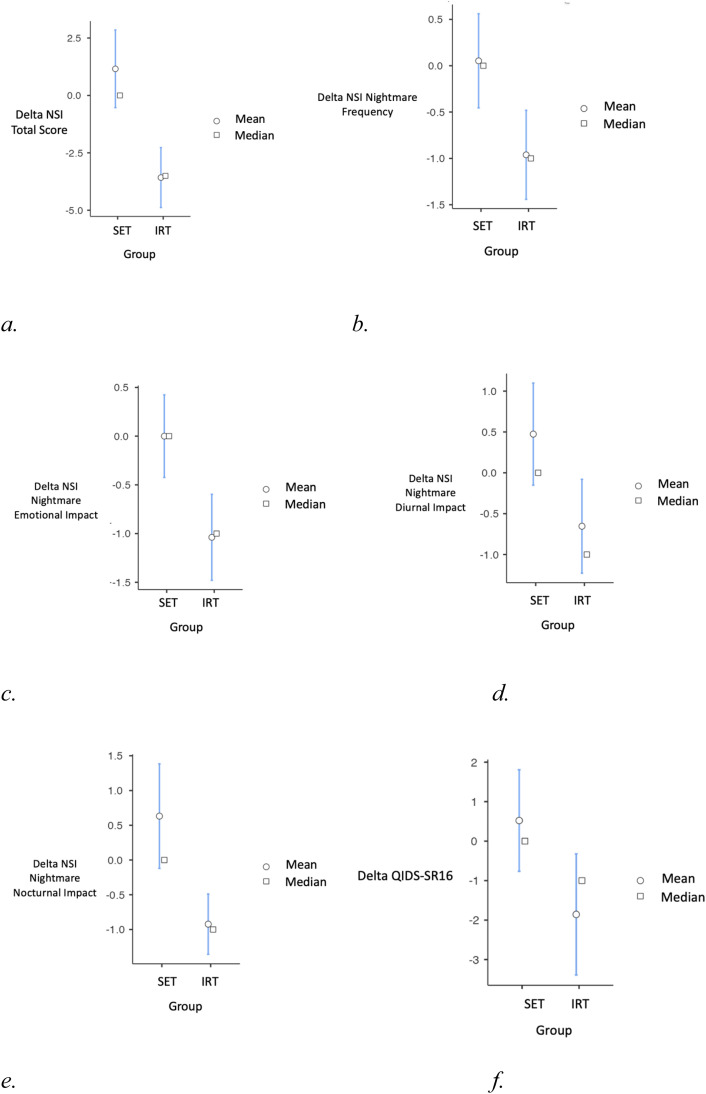

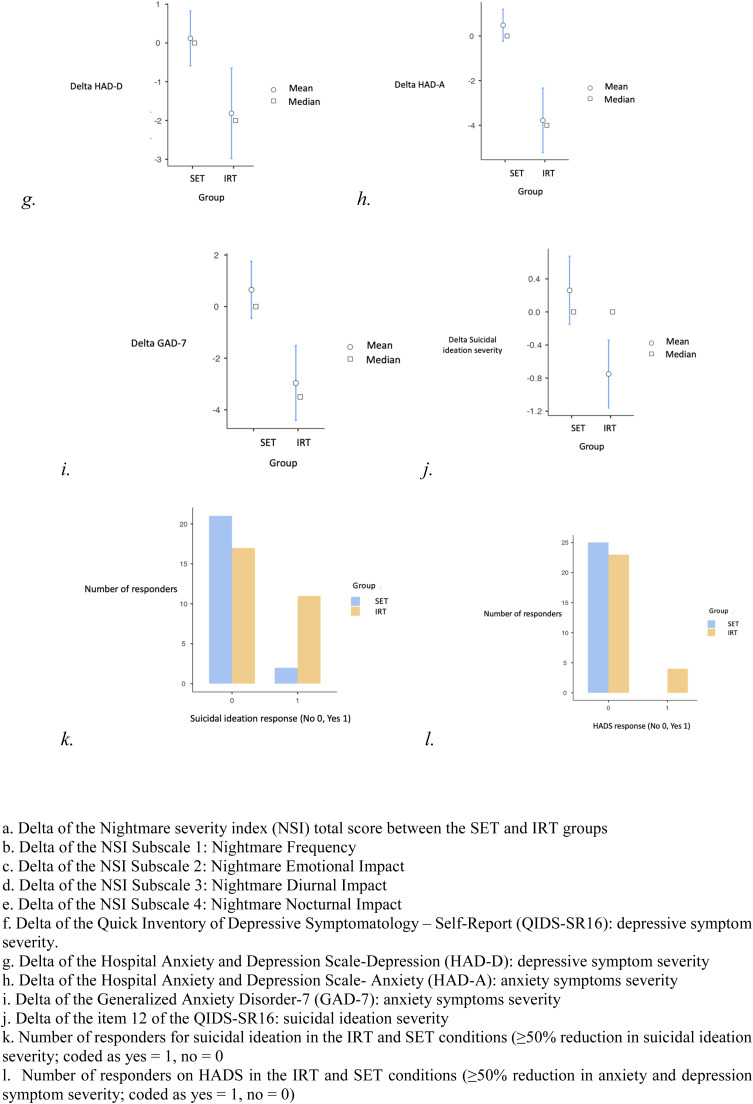
Difference in scores (post-group minus pre-group) for the Sleep education therapy (SET) and Imagery Rehearsal Therapy (IRT) groups. a. Delta of the Nightmare severity index (NSI) total score between the SET and IRT groups, b. Delta of the NSI Subscale 1: Nightmare Frequency, c. Delta of the NSI Subscale 2: Nightmare Emotional Impact, d. Delta of the NSI Subscale 3: Nightmare Diurnal Impact, e. Delta of the NSI Subscale 4: Nightmare Nocturnal Impact, f. Delta of the Quick Inventory of Depressive Symptomatology – Self-Report (QIDS-SR16): depressive symptom severity., g. Delta of the Hospital Anxiety and Depression Scale-Depression (HAD-D): depressive symptom severity, h. Delta of the Hospital Anxiety and Depression Scale- Anxiety (HAD-A): anxiety symptoms severity, i. Delta of the Generalized Anxiety Disorder-7 (GAD-7): anxiety symptoms severity, j. Delta of the item 12 of the QIDS-SR16: suicidal ideation severity, k. Number of responders for suicidal ideation in the IRT and SET conditions (≥50 % reduction in suicidal ideation severity; coded as yes = 1, no = 0, l. Number of responders on HADS in the IRT and SET conditions (≥50 % reduction in anxiety and depression symptom severity; coded as yes = 1, no = 0).

We then analysed the difference from baseline to posttreatment in each group and compared the difference from baseline to post-treatment between the two groups on nightmares and sleep-related characteristics (Table 2, Fig. 1). First, the improvement in nightmare severity measured by the NSI was significantly higher in the IRT group compared to the SET group (*p* < 0.001 and Cohen's *d* = 1.3326), as well as for all its subdimensions: nightmares frequency (*p* = 0.010, *d* = 0.8452), emotional impact (*p* = 0.003, *d* = 0.973), diurnal impact (*p* = 0.017, *d* = 0.777), and nocturnal impact (*p* = 0.002, *d* = 1.125). These p-values remained statistically significant after applying the Benjamini-Hochberg correction for multiple comparisons. The response (50 % reduction in NSI score from baseline) was not significantly different between the two groups (*p* = 0.073). The 33 % response rate, however, was significantly higher in the IRT group compared to the control group (*p* = 0.004) along with the 25 % one (*p* < 0001). Regarding sleep characteristics, improvement was significantly higher in the IRT group than in the control group for ISI5 (*p* = 0.005, *d* = 0.7841), PSQI total score (*p* = 0.038, *d* = 0.4074), PSQI sleep duration (*p* = 0.033, *d* = 0.57), and PSQI sleep efficiency (*p* = 0.022, *d* = 0.60). ISI5 corresponded to worrying about sleep difficulties. Regarding the others sleep secondaries criteria, there were no significant group differences.

### IRT effects on depressive symptoms and suicidal ideation

Table 3 summarizes the effect of IRT on depressive symptoms, anxiety and suicidal ideation (Fig. 1). Firstly, the primary evaluation criterion for depressive symptoms, ΔHAD-D showed significantly greater improvement in the IRT group compared to the SET group (*p* < 0.001 and Cohen's *d* = 0.755), as well as the 50 % (*p* = 0.003) and 25 % responses (*p* < 0.001). Additionally, the improvement in suicidal ideation (*p* = 0.002, *d* = 0.948) (item 12 of QIDS-SR16) as well as the response (*p* = 0.013) were also stronger in the IRT group compared to the SET group. All other secondary scores for anxiety symptoms (ΔGAD-7 *p* < 0.001, *d* = 1.074 and ΔHAD-A *p* < 0.001, *d* = 1.402), depressive symptoms (ΔQIDS-SR16 *p* = 0.028, *d* = 0.638), and anxiety-depressive symptoms (ΔHAD *p* < 0.001, *d* = 1.251 and HADS response *p* = 0.045) exhibited significantly greater improvement in the IRT compared to the SET group.

### Multivariate analysis adjustment for IRT effects

To address the sample differences in PSQI Sleep latency at baseline, we conducted multivariate analysis to determine if the observed effects of IRT were attributable to this difference (Table 4). All differences observed in the improvement of tests in the IRT group remained significant after accounting for the sample differences in PSQI sleep latency. The IRT effects were therefore not due to sample differences.

**Table 4:** Multivariate analysis for baseline clinical characteristic differences between the Imagery Rehearsal Therapy (IRT) and Sleep Education Therapy (SET) groups.

	p	p*
ΔNSI - Nightmares Severity	<0,001	<0,001
ΔNSI SS1 (Frequency)	0,010	0,015
ΔNSI SS2 (Emotional impact)	0,003	0,009
ΔNSI SS3 (Diurnal impact)	0,017	0,041
ΔNSI SS4 (Nocturnal impact)	0,002	<0,001
ΔHAD	<0,001	<0,001
ΔHAD-D	<0,001	0,019
ΔHAD-A	<0,001	<0,001
ΔGAD-7	<0,001	0,001
ΔQIDS-SR16	0,028	0,022
ΔSuicidal Ideation	0,002	0,001

p.

⁎ ajustement for PSQI Sleep latency NSI=Night severity index; GAD-7= Generalized Anxiety Disorder-7; HAD=Hospital anxiety and depression scale; QIDS-SR16=Quick inventory depressive symptoms-self report 16 items.

**Table 5:** Multivariate analyses to identify factors predicting improvement in Nightmare Severity Index (ΔNSI). (NSI=Nightmare severity index; MDE=Major depressive disorder; TRD=Treatment resistant-depression).

NSI IMPROVEMENT	Estimate (ß)	SE(ß)	Z	p
MDE with Anxiety	2,1315	2,233	0,955	0,092
MDE with TRD	−2,7437	2,917	−0,941	0,308

### Linear mixed-effects model results

For the primary outcome (Nightmare Severity Index, NSI), the mixed-effects model revealed a statistically significant time × group interaction (β = 4.73, *p* < 0.001), indicating greater improvement in NSI scores for the IRT group compared to the SET group over time

(Supplementary Table 1). These results were consistent with our original analyses. Significant time × group interactions were observed across all four NSI subscales, indicating greater improvements in the IRT group compared to the SET group (NSI SS1 – Nightmare Frequency: β = 1.145, 95 % CI [0.427, 1.864], *p* = 0.003; NSI SS2 – Emotional Impact β = 1.038, 95 % CI [0.416, 1.660], *p* = 0.002; NSI SS3 – Diurnal Impact: β = 1.128, 95 % CI [0.265, 1.990], *p* = 0.013; NSI SS4 – Nocturnal Impact β = 1.555, 95 % CI [0.672, 2.438], *p* = 0.001) (Supplementary Tables 2 to 5). These results indicate that IRT led to significantly greater reductions in nightmare severity across multiple domains compared to the control condition (SET). Significant time × group interactions were observed across multiple clinical outcomes, indicating greater improvements in the IRT group compared to the SET group: (HAD-D – Depressive Symptoms β = 1.998, 95 % CI [0.612, 3.384], *p* = 0.006; HAD-A – Anxiety Symptoms β = 4.34, 95 % CI [2.710, 5.966], *p* < 0.001; HAD – Combined Anxiety and Depressive Symptoms β = 6.36, 95 % CI [3.70, 9.02], *p* < 0.001; GAD-7 β = 3.57, 95 % CI [1.717, 5.414], *p* < 0.001; QIDS-SR16 Total β = 2.412, 95 % CI [0.394, 4.430], *p* = 0.021; Suicidal Ideation β = 0.592, 95 % CI [0.071, 1.113], *p* = 0.029) (Supplementary Tables 6 to 11). These findings indicate that IRT led to significantly greater reductions in anxiety, depressive symptoms, and suicidal ideation compared to the control condition (SET) over time.

### Predictive factors of improvement in nightmares

Finally, we aimed to identify predictive factors for improvement in nightmares using the NSI and its sub-scores. After the univariate analysis (supplementary Tables 12 to 18), we found that MDE with TRD was a significant positive predictor (*p* = 0.007) of improvement in the emotional impact of nightmares (ΔNSI SS2) following an IRT intervention (Table 6), while a high frequency of nightmares was a significant negative predictor (*p* = 0.008) of improvement in the emotional impact of nightmares (ΔNSI SS2) following an IRT intervention. Moreover, daytime dysfunction measured by the PSQI was a good predictor of reduction in insomnia (ΔNSI SS4) related to nightmares (*p* = 0.008) (Table 7).

**Table 6:** Multivariate analyses to identify factors predicting improvement in Nightmare Severity Index (NSI) Subscale 2 Nightmare Emotional Impact (ΔNSI SS2). (NSI=Nightmare severity index; MDE=Major depressive disorder; TRD=Treatment resistant-depression).

NSI SS2 IMPROVEMENT	Estimate (ß)	SE(ß)	Z	p
MDE with TRD	−1,438	0,474	−3,03	0,007
NSI-SS1	0,365	0,122	2,99	0,008

**Table 7:** Multivariate analyses to identify factors predicting improvement in Nightmare Severity Index (NSI) Subscale 4 Nightmare Nocturnal Impact (ΔNSI SS4). (NSI=Nightmare severity index; PSQI=Pittsburgh sleep quality index).

NSI SS4 IMPROVEMENT	Estimate (ß)	SE(ß)	Z	p
PSQI Daytime dysfunction	−0,683	0,234	−2,913	0,008

No factor was found to be a significant predictor of overall nightmare improvement (ΔNSI), nightmare response (33 % and 25 %), reduction in the frequency of nightmares (ΔNSI SS1), or improvement in the diurnal impact of nightmares (ΔNSI SS3) following an IRT intervention.

## Discussion

This first non-randomized controlled study examined the association between Imagery Rehearsal Therapy (IRT) and clinical outcomes in patients with MDE and Nightmare Disorder. IRT compared to SET was associated with a greater reduction in nightmare disorder severity, improving all dimensions: nightmares frequency, nocturnal impact, diurnal impact, and emotional impact. In addition, IRT compared to SET was significantly associated with improvements in depressive symptoms, anxious symptoms, and suicidal ideation, not only showing improvement but also a response with a decrease of 50 % in both suicidal ideation and a reduction in the severity of clinical anxious symptoms and depressive symptoms. All improvements consistently yield clinically relevant effect sizes: 0.755 for depressive symptoms HAD-D, 1.402 for anxious symptoms HAD-A, 1.3326 for NSI, and 0.948 for suicidal ideation. Moreover, among other predictors, treatment-resistant depressions predicted a greater reduction in the emotional impact of nightmares, whereas high frequency of nightmares predicted the opposite. Daytime dysfunction predicted a greater reduction in difficulty failing asleep and in sleep continuity alteration (ΔNSI SS4) related to nightmares. Furthermore, the use of hypnotic, antidepressant, and mood-stabilizing medications did not predict a poorer response to IRT.

This study aligns with and validates the preliminary study by Ellis et al. (2019), which through a case-series design showed that IRT could effectively reduce suicidal ideation among patients with psychiatric disorder including depression. It also validates that depressive symptoms were reduced in a population with MDE (Thünker & Pietrowsky, 2012). Additionally, this study extends previous research in a controlled manner previous studies by showing that IRT is associated with a with a reduction in nightmares, as measured by the NSI scale and all its sub-dimensions, thereby clarifying the effects of IRT on nightmare frequency, as well as emotional, nocturnal, and diurnal impact. While previous studies highlighted IRT's impact on nightmare frequency and anxiety within psychiatric populations (van Schagen et al., 2015), our research shows broader improvements across all subdimensions of nightmare experience as measured by the NSI. Moreover, this expands the range of psychiatric populations that may benefit from IRT, supporting preliminary evidence of its efficacy for nightmares in patients with MDE, as reported by Thunker et al. in 2012.

Our study also demonstrated that IRT was associated with improvements in sleep quality, particularly sleep efficiency and duration, as well as sleep-related worry, which aligns with the apprehension and avoidance dimension identified in chronic nightmares (Werner et al., 2021). However, although the nocturnal impact was significantly diminished in the IRT group, overall insomnia, as measured by ISI, did not show a significant decrease, suggesting that IRT specifically targeted certain dimensions of sleep, such as sleep efficacy, rather than insomnia per se.

The potential mechanism by which Imagery Rehearsal Therapy (IRT) reduces suicidality in patients with MDE and Nightmare Disorder appears to be multifactorial. First, IRT significantly reduces the emotional impact of nightmares—a factor more strongly correlated with depression and suicidal ideation than nightmare frequency alone (Ellis et al., 2019). By transforming the distressing emotional content of nightmares through cognitive restructuring and positive imagery, IRT alleviates the nocturnal emotional burden that fuels daytime hopelessness and despair. Second, IRT leads to substantial improvements in depressive symptoms, which are among the strongest predictors of suicidal ideation (Thünker & Pietrowsky, 2012). By alleviating core depressive features such as anhedonia, guilt, and negative thought patterns, IRT may reduce the cognitive and emotional vulnerability to suicide. Third, the therapy also reduces anxious symptoms, which frequently co-occur with depression and independently contribute to suicidal risk. Fourth, IRT improves sleep quality—including sleep efficiency, duration, and a reduction in sleep-related worry—thereby targeting a major and often underestimated suicide risk factor: sleep disturbance (Ellis et al., 2019). Poor sleep intensifies emotional dysregulation, rumination, and cognitive distortions, all of which are key drivers of suicidal thinking. By acting on these interconnected pathways—depression, anxiety, emotional distress, and sleep dysfunction—IRT may exert a protective effect against the onset or escalation of suicidal ideation. These findings support the idea that IRT is not merely a treatment for nightmares, but a potentially valuable intervention in reducing suicide risk in vulnerable depressive populations.

Surprisingly, treatment-resistant depression seemed to be a predictive factor of IRT effectiveness, a hopeful finding given the importance of having an effective therapy for treatment-resistant depression associated with high suicide risk. Understanding the mechanism underlying the effectiveness of IRT in cases of MDE with TRD and nightmare disorder is of paramount importance for clinical practice. Patients suffering from MDE and TRD experience insufficient responses to conventional treatments such as antidepressants and psychotherapies. By integrating IRT into the treatment of treatment-resistant depression with nightmare disorder, IRT could open new perspectives and greatly enhance the quality of life for these vulnerable patients while improving their response to drugs.

This study has several limitations. Although it benefited from a first-time controlled design and a structured therapeutic framework, it remains an exploratory study with a limited sample size and a short timeline.

It is necessary to conduct a long-term randomized controlled study, particularly to evaluate the effect of IRT on the incidence of suicidal crises and to determine the causality of nightmare treatment in preventing the conversion of depressive symptoms into suicidal crises by halting the progression of nightmare content alteration. Another limitation lies in the absence of long-term follow-up, which prevents us from determining whether the benefits of IRT are sustained over time. In addition, we acknowledge the limitation of using a single-item scale and suggest future studies adopt more comprehensive tools such as the Columbia-Suicide Severity Rating Scale (C-SSRS) (Beck et al., 1999; Posner et al., 2011; Quevedo-Blasco et al., 2023). SET control group received one session versus four sessions of IRT. This may introduce a non-specific "dose" and attention bias. Future studies should consider a dose-matched active control (e.g., 4 sessions of sleep education or imagery exposure). Nevertheless, the SET group represents an active and meaningful comparison condition rather than a passive waiting-list control. Moreover, as with all psychotherapeutic interventions, non-specific therapeutic factors such as group support, therapeutic alliance, and participants' expectations may have contributed to symptom improvement independently of the IRT technique itself (Stern et al., 2024). These factors should be carefully considered and controlled for in future studies. A key further limitation of the present study lies in its data collection design, which included only two time points: baseline and post-intervention assessments. Including a longer-term follow-up would have been valuable for evaluating the maintenance and durability of treatment effects over time. Such longitudinal data, combined with a larger sample size, would provide a better opportunity to use mixed-effects models, offering a more comprehensive understanding of the long-term impact of IRT on nightmare severity and related psychological symptoms.

For a comprehensive assessment of nightmare disorder and MDE, and to advocate for a more individualized biosignature approach, it would be advisable to complement our method with additional diagnostic tools. Analysing correlations between improvements in depressive symptoms, suicidality, and objective sleep characteristics from actigraphy and polysomnographic measurements would be insightful (Ricka et al., 2023). Likewise, research examining the neurological correlates of improvements in nightmares and mood disorders could enhance our understanding of the pathophysiological mechanisms underlying the reduction of depressive symptoms and suicidal ideation. This is particularly relevant considering that nightmare disorder and depression share a part of their neurobiological basis in the limbic system (Levin & Nielsen, 2007).

## Conclusion

In conclusion, this study found that IRT, compared to SERT, was associated with improvements in overall nightmare severity and across all its dimensions—frequency, nocturnal impact, diurnal impact, and emotional impact—in patients with MDE. Additionally, IRT was linked to clinically meaningful reductions in depressive symptoms, anxiety, and suicidal ideation in this population. Improvements were also observed in sleep efficiency, duration, and insomnia-related concerns. While these findings are promising, they should be considered preliminary. Further replication through larger, randomized controlled trials is needed to better understand the clinical relevance and therapeutic role of IRT in individuals with MDE.

## Authorship contribution statement

J. Maruani, N. Martins and P.A. Geoffroy: Writing - original draft. made the statistical analyses. All others authors: Validation, Visualization, Writing - review & editing. All authors contributed to the article and approved the submitted version.

## Data availability statement

All data generated or analyzed during this study are included in this article and its supplementary material files. Further enquiries can be directed to the corresponding author.

## Statement of ethics

This project is part of the "Som-Psy" routine care cohort: Study of sleep and circadian rhythms biomarkers in psychiatric disorders promoted by Assistance Publique - Hôpitaux de Paris with the favorable opinion (No. CER-2020–56) of the Committee for Evaluation of the Ethics of Biomedical Research Projects (CEERB) Paris Nord (Institutional Review Board - IRB 00,006,477- of the HUPNVS, Paris 7 University, AP-HP). Patients have received a non-opposition information note regarding the purpose of this study and that clinical information may be used for research.

## Statement

During the preparation of this work, we used generative AI technologies to edit the English text.

## Declaration of competing interest

no biomedical financial interests or potential conflicts of interest related to the article.

## Appendix Supplementary materials

Image, application 1
