# Supplementary material for: Imagery Rehearsal Therapy (IRT) is associated with reduced nightmare severity and depressive, anxiety and suicidal symptoms in adults with Major Depressive Episode
Source: Int J Clin Health Psychol. 2025 Dec 15;26(1):100658. doi: 10.1016/j.ijchp.2025.100658 (PMC12767828; doi:10.1016/j.ijchp.2025.100658)
Supplement: Supplementary file 1 [file mmc1.docx]

Supplementary Table 1: Linear Mixed-Effects Model Results for Nightmare Severity Index (NSI) Total Score: Imagery Rehearsal Therapy (IRT) versus Sleep Education Therapy (SET) Group over Time (Pre- and Post-Intervention, 0 = Pre, 1 = Post)

| Parameter Estimates (Fixed coefficients) | | | | | | | | |
| --- | --- | --- | --- | --- | --- | --- | --- | --- |
|  | | | | **95% Confidence Intervals** | |  | | |
| **Names** | **Effect** | **Estimate** | **SE** | **Lower** | **Upper** | **df** | **t** | **p** |
| (Intercept) | (Intercept) | 13.83 | 0.521 | 12.792 | 14.869 | 42.1 | 26.52 | <.001 |
| Time | 0 - 1 | 1.21 | 0.545 | 0.124 | 2.295 | 36.5 | 2.22 | 0.033 |
| Group | IRT - SET | -1.39 | 1.043 | -3.469 | 0.686 | 42.1 | -1.33 | 0.189 |
| Time ✻ Group | (0 - 1) ✻ (IRT - SET) | **4.73** | 1.090 | 2.564 | 6.906 | 36.5 | 4.34 | **<.001** |

Supplementary Table 2: Linear Mixed-Effects Model Results for Nightmare Severity Index (NSI) Subscale 1 Nightmare Frequency: Imagery Rehearsal Therapy (IRT) versus Sleep Education Therapy (SET) Group over Time (Pre- and Post-Intervention, 0 = Pre, 1 = Post)

Parameter Estimates (Fixed coefficients)

|  | | | | **95% Confidence Intervals** | |  | | |
| --- | --- | --- | --- | --- | --- | --- | --- | --- |
| **Names** | **Effect** | **Estimate** | **SE** | **Lower** | **Upper** | **df** | **t** | **p** |
| (Intercept) | (Intercept) | 2.944 | 0.190 | 2.5647 | 3.322 | 41.6 | 15.47 | <.001 |
| Group | IRT - SET | -0.429 | 0.381 | -1.1870 | 0.328 | 41.6 | -1.13 | 0.266 |
| Time | 0 - 1 | 0.456 | 0.180 | 0.0972 | 0.815 | 38.4 | 2.53 | 0.016 |
| Group ✻ Time | (IRT - SET) ✻ (0 - 1) | **1.145** | 0.361 | 0.4272 | 1.864 | 38.4 | 3.18 | **0.003** |

Supplementary Table 3: Linear Mixed-Effects Model Results for Nightmare Severity Index (NSI) Subscale 2 Nightmare Emotional Impact: Imagery Rehearsal Therapy (IRT) versus Sleep Education Therapy (SET) Group over Time (Pre- and Post-Intervention, 0 = Pre, 1 = Post)

| Parameter Estimates (Fixed coefficients) | | | | | | | | |
| --- | --- | --- | --- | --- | --- | --- | --- | --- |
|  | | | | **95% Confidence Intervals** | |  | | |
| **Names** | **Effect** | **Estimate** | **SE** | **Lower** | **Upper** | **df** | **t** | **p** |
| (Intercept) | (Intercept) | 3.882 | 0.143 | 3.596 | 4.168 | 40.9 | 27.06 | <.001 |
| Time | 0 - 1 | 0.519 | 0.156 | 0.208 | 0.830 | 42.3 | 3.33 | 0.002 |
| Group | IRT - SET | -0.341 | 0.287 | -0.913 | 0.230 | 40.9 | -1.19 | 0.241 |
| Time ✻ Group | (0 - 1) ✻ (IRT - SET) | **1.038** | 0.312 | 0.416 | 1.660 | 42.3 | 3.33 | **0.002** |

Supplementary Table 4: Linear Mixed-Effects Model Results for Nightmare Severity Index (NSI) Subscale 3 Nightmare Diurnal Impact: Imagery Rehearsal Therapy (IRT) versus Sleep Education Therapy (SET) Group over Time (Pre- and Post-Intervention, 0 = Pre, 1 = Post)

| Parameter Estimates (Fixed coefficients) | | | | | | | | |
| --- | --- | --- | --- | --- | --- | --- | --- | --- |
|  | | | | **95% Confidence Intervals** | |  | | |
| **Names** | **Effect** | **Estimate** | **SE** | **Lower** | **Upper** | **df** | **t** | **p** |
| (Intercept) | (Intercept) | 3.4013 | 0.165 | 3.072 | 3.731 | 40.8 | 20.578 | <.001 |
| Time | 0 - 1 | 0.0901 | 0.217 | -0.341 | 0.521 | 40.5 | 0.416 | 0.680 |
| Group | IRT - SET | -0.3026 | 0.331 | -0.961 | 0.356 | 40.8 | -0.915 | 0.365 |
| Time ✻ Group | (0 - 1) ✻ (IRT - SET) | **1.1275** | 0.433 | 0.265 | 1.990 | 40.5 | 2.604 | **0.013** |

Supplementary Table 5: Linear Mixed-Effects Model Results for Nightmare Severity Index (NSI) Subscale 4 Nightmare Nocturnal Impact: Imagery Rehearsal Therapy (IRT) versus Sleep Education Therapy (SET) Group over Time (Pre- and Post-Intervention, 0 = Pre, 1 = Post)

| Parameter Estimates (Fixed coefficients) | | | | | | | | |
| --- | --- | --- | --- | --- | --- | --- | --- | --- |
|  | | | | **95% Confidence Intervals** | |  | | |
| **Names** | **Effect** | **Estimate** | **SE** | **Lower** | **Upper** | **df** | **t** | **p** |
| (Intercept) | (Intercept) | 3.470 | 0.164 | 3.143 | 3.796 | 39.3 | 21.175 | <.001 |
| Time | 0 - 1 | 0.146 | 0.222 | -0.296 | 0.587 | 29.6 | 0.658 | 0.516 |
| Group | IRT - SET | -0.324 | 0.328 | -0.977 | 0.329 | 39.3 | -0.988 | 0.329 |
| Time ✻ Group | (0 - 1) ✻ (IRT - SET) | **1.555** | 0.443 | 0.672 | 2.438 | 29.6 | 3.508 | **0.001** |
|  | | | | | | | | |

Supplementary Table 6: Linear Mixed-Effects Model Results for Hospital Anxiety and Depression Scale (HAD-S): Imagery Rehearsal Therapy (IRT) versus Sleep Education Therapy (SET) Group over Time (Pre- and Post-Intervention, 0 = Pre, 1 = Post)

| Parameter Estimates (Fixed coefficients) | | | | | | | | |
| --- | --- | --- | --- | --- | --- | --- | --- | --- |
|  | | | | **95% Confidence Intervals** | |  | | |
| **Names** | **Effect** | **Estimate** | **SE** | **Lower** | **Upper** | **df** | **t** | **p** |
| (Intercept) | (Intercept) | 19.77 | 0.922 | 17.94 | 21.61 | 49.4 | 21.45 | <.001 |
| Time | 0 - 1 | 2.58 | 0.670 | 1.25 | 3.91 | 37.3 | 3.85 | <.001 |
| Groupe | IRT - SET | -2.57 | 1.844 | -6.23 | 1.09 | 49.4 | -1.40 | 0.169 |
| Time ✻ Groupe | (0 - 1) ✻ (IRT - SET) | **6.36** | 1.340 | 3.70 | 9.02 | 37.3 | 4.75 | **<.001** |

Supplementary Table 7: Linear Mixed-Effects Model Results for Hospital Anxiety and Depression Scale- Anxiety (HAD-A): Imagery Rehearsal Therapy (IRT) versus Sleep Education Therapy (SET) Group over Time (Pre- and Post-Intervention, 0 = Pre, 1 = Post)

| Parameter Estimates (Fixed coefficients) | | | | | | | | |
| --- | --- | --- | --- | --- | --- | --- | --- | --- |
|  | | | | **95% Confidence Intervals** | |  | | |
| **Names** | **Effect** | **Estimate** | **SE** | **Lower** | **Upper** | **df** | **t** | **p** |
| (Intercept) | (Intercept) | 11.64 | 0.569 | 10.515 | 12.774 | 50.0 | 20.48 | <.001 |
| Time | 0 - 1 | 1.69 | 0.410 | 0.875 | 2.503 | 38.6 | 4.12 | <.001 |
| Groupe | IRT - SET | -1.43 | 1.137 | -3.690 | 0.828 | 50.0 | -1.26 | 0.214 |
| Time ✻ Group | (0 - 1) ✻ (IRT - SET | **4.34** | 0.819 | 2.710 | 5.966 | 38.6 | 5.30 | **<.001** |

Supplementary Table 8: Linear Mixed-Effects Model Results for Hospital Anxiety and Depression Scale- Depression (HAD-D): Imagery Rehearsal Therapy (IRT) versus Sleep Education Therapy (SET) Group over Time (Pre- and Post-Intervention, 0 = Pre, 1 = Post)

| Parameter Estimates (Fixed coefficients) | | | | | | | | |
| --- | --- | --- | --- | --- | --- | --- | --- | --- |
|  | | | | **95% Confidence Intervals** | |  | | |
| **Names** | **Effect** | **Estimate** | **SE** | **Lower** | **Upper** | **df** | **t** | **p** |
| (Intercept) | (Intercept) | 8.123 | 0.557 | 7.017 | 9.23 | 49.8 | 14.59 | <.001 |
| Time | 0 - 1 | 0.879 | 0.349 | 0.186 | 1.57 | 42.6 | 2.52 | 0.016 |
| Group | RIM - SET | -1.154 | 1.114 | -3.366 | 1.06 | 49.8 | -1.04 | 0.305 |
| Time ✻ Group | (0 - 1) ✻ (IRT - SET | **1.998** | 0.698 | 0.612 | 3.38 | 42.6 | 2.86 | **0.006** |

Supplementary Table 9: Linear Mixed-Effects Model Results for Generalized Anxiety Disorder-7 (GAD-7): Imagery Rehearsal Therapy (IRT) versus Sleep Education Therapy (SET) Group over Time (Pre- and Post-Intervention, 0 = Pre, 1 = Post)

| Parameter Estimates (Fixed coefficients) | | | | | | | | |
| --- | --- | --- | --- | --- | --- | --- | --- | --- |
|  | | | | **95% Confidence Intervals** | |  | | |
| **Names** | **Effect** | **Estimate** | **SE** | **Lower** | **Upper** | **df** | **t** | **p** |
| (Intercept) | (Intercept) | 11.18 | 0.719 | 9.749 | 12.605 | 49.5 | 15.56 | <.001 |
| Time | 0 - 1 | 1.18 | 0.465 | 0.258 | 2.106 | 47.8 | 2.54 | 0.014 |
| Group | IRT - SET | -1.96 | 1.437 | -4.817 | 0.894 | 49.5 | -1.36 | 0.178 |
| Time ✻ Group | (0 - 1) ✻ (IRT - SET) | **3.57** | 0.930 | 1.717 | 5.414 | 47.8 | 3.83 | **<.001** |

Supplementary Table 10: Linear Mixed-Effects Model Results for Quick Inventory of Depressive Symptomatology – Self-Report (QIDS-SR16): Imagery Rehearsal Therapy (IRT) versus Sleep Education Therapy (SET) Group Over Time (Pre- and Post-Intervention, 0 = Pre, 1 = Post)

| Parameter Estimates (Fixed coefficients) | | | | | | | | |
| --- | --- | --- | --- | --- | --- | --- | --- | --- |
|  | | | | **95% Confidence Intervals** | |  | | |
| **Names** | **Effect** | **Estimate** | **SE** | **Lower** | **Upper** | **df** | **t** | **p** |
| (Intercept) | (Intercept) | 12.534 | 0.662 | 11.219 | 13.849 | 47.5 | 18.94 | <.001 |
| Time | 0 - 1 | 0.651 | 0.508 | -0.358 | 1.660 | 49.3 | 1.28 | 0.206 |
| Group | IRT - SET | -1.711 | 1.324 | -4.341 | 0.920 | 47.5 | -1.29 | 0.203 |
| Time ✻ Group | (0 - 1) ✻ (IRT- SET) | **2.412** | 1.015 | 0.394 | 4.430 | 49.3 | 2.38 | **0.021** |

Supplementary Table 11: Linear Mixed-Effects Model Results for Suicidal Ideation (QIDS-SR16 Item 12): Imagery Rehearsal Therapy (IRT) versus Sleep Education Therapy (SET) Group Over Time (Pre- and Post-Intervention, 0 = Pre, 1 = Post)

| Parameter Estimates (Fixed coefficients) | | | | | | | | |
| --- | --- | --- | --- | --- | --- | --- | --- | --- |
|  | | | | **95% Confidence Intervals** | |  | | |
| **Names** | **Effect** | **Estimate** | **SE** | **Lower** | **Upper** | **df** | **t** | **p** |
| (Intercept) | (Intercept) | 0.5811 | 0.0995 | 0.3834 | 0.779 | 50.0 | 5.8416 | <.001 |
| Time | 0 - 1 | 0.0253 | 0.1311 | -0.2352 | 0.286 | 46.1 | 0.1933 | 0.848 |
| Group | IRT - SET | 0.0163 | 0.1990 | -0.3791 | 0.412 | 50.0 | 0.0821 | 0.935 |
| Time ✻ Group | (0 - 1) ✻ (IRT – SET) | **0.5922** | 0.2622 | 0.0711 | 1.113 | 46.1 | 2.2586 | **0.029** |

## Supplementary Table 12: Univariate analyses to identify factors predicting improvement in Nightmare Severity Index (ΔNSI).

*(NSI=Nightmare severity index ; ISI=Insomnia severity index ; ESS=Epworth sleepiness scale ; PSQI=Pittsburgh sleep quality index ; CES-D=Center for Epidemiologic Studies Depression Scale ; GAD-7= Generalized Anxiety Disorder-7; HAD=Hospital anxiety and depression scale ; QIDS-SR16=Quick inventory depressive symptoms-self report 16 items ; SUD=Substance use disorder ; MDE=Major depressive disorder ; TRD=Treatment resistant-depression ; MADRE=The Mannheim dream questionnaire ; PTSD=Post-Traumatic stress disorder ; GAD=Generalized Anxiety Disorder ; MADRS=Montgomery and Asberg Depression Rating Scale)*

|  | **Estimate (ß)** | **SE(ß)** | **Z** | **p** |
| --- | --- | --- | --- | --- |
| ***NSI improvement*** | | | | |
| **Age** | -0,0535 | 0,0435 | -1,229 | 0,231 |
| **Gender** | 0,307 | 1,43 | 0,215 | 0,831 |
| ***Sleep parameters*** | | | | |
| **PSQI Sleep duration** | 0,201 | 0,536 | 0,374 | 0,711 |
| **PSQI Sleep subjective quality** | ^-0,906^ | 0,880 | ^-1,030^ | 0,314 |
| **PSQI sleep latency** | 0,195 | 0,717 | 0,273 | 0,788 |
| **PSQI Daytime dysfunction** | -1,05 | 0,779 | -1,351 | 0,191 |
| **PSQI Sleep efficiency** | 0,255 | 0,568 | 0,449 | 0,657 |
| **PSQI Sleep disturbance** | -0,616 | 1,17 | -0,526 | 0,604 |
| **PSQI Sleep promoting medication** | -0,403 | 0,521 | -0,826 | 0,418 |
| **PSQI** | -0,0761 | 0,213 | -0,357 | 0,725 |
| **ESS** | -0,0779 | 0,143 | -0,543 | 0,592 |
| **Horne and Ostberg** | -0,0806 | 0,0844 | -0,9555 | 0,349 |
| **NSI – Nightmares Severity** | -0,133 | 0,188 | -0,707 | 0,487 |
| **NSI SS1 Frequency** | 0,1 | 0,481 | 0,208 | 0,837 |
| **NSI SS2 Emotional Impact** | 0,459 | 0,758 | 0,606 | 0,550 |
| **NSI SS3 Diurnal Impact** | -0,703 | 0,527 | -1,334 | 0,195 |
| **NSI SS4 Nocturnal Impact** | -0,789 | 0,534 | -1,477 | 0,153 |
| **ISI Total score** | -0,0699 | 0,141 | -0,494 | 0,626 |
| **ISI1a - Severity of sleep latency** | -0,274 | 0,607 | -0,451 | 0,656 |
| **ISI1b - Difficulty staying asleep** | -0,501 | 0,564 | -0,889 | 0,383 |
| **ISI1c - Early morning awakening** | -0,698 | 0,472 | -1,48 | 0,152 |
| **ISI2 - Satisfaction with current sleep** | 0,0934 | 0,755 | 0n124 | 0,923 |
| **ISI3 - Disruption of daily functioning** | 0,591 | 0,649 | 0,911 | 0,372 |
| **ISI4 - Apparent difficulties** | 0,218 | 0,566 | 0,386 | 0,703 |
| **ISI5 - Concerns regarding sleep disturbances** | -0,349 | 0,805 | -0,433 | 0,669 |
| ***Mood parameters*** | | | | |
| **MDE with anxiety** | 3,42 | 1,343 | 2,55 | **0,021** |
| **MDE with seasonal feature** | 0,3 | 1,788 | 0,168 | 0,869 |
| **MDE with TRD** | -4,17 | 1,947 | -2,14 | **0,046** |
| ***Severity of Depression and anxiety*** | | | | |
| **GAD-7** | 0,219 | 0,102 | 2,14 | **0,043** |
| **HAD** | 0,0699 | 0,102 | 0,682 | 0,502 |
| **HAD-D** | 0,0186 | 0,172 | 0,108 | 0,915 |
| **HAD-A** | 0,164 | 0,163 | 1,00 | 0,326 |
| ***SUD*** | | | | |
| **SUD** | -0,694 | 1,807 | -0,384 | 0,705 |
| **Current tobacco smoking** | -0,867 | 1,528 | -0,567 | 0,577 |
| **Current alcohol use disorder** | -1,45 | 2,411 | -0,601 | 0,554 |
| **Current cannabis use disorder** | 3,33 | 3,274 | 1,02 | 0,321 |
| ***Treatments*** | | | | |
| **Antidepressants** | -0,385 | 2,2 | -0,175 | 0,864 |
| **Anxiolytics** | 0,557 | 1,66 | 0,335 | 0,742 |
| **Mood stabilizers** | -0,974 | 1,917 | -0,508 | 0,619 |
| **Hypnotics** | 0,557 | 1,66 | 0,335 | 0,742 |
| **Antipsychotics** | -1,05 | 2,139 | -0,49 | 0,631 |
| ***Comorbidities*** | | | | |
| **PTSD** | -1,51 | 1,697 | -0,892 | 0,381 |
| **GAD** | 0,219 | 1,724 | 0,127 | 0,900 |

## Supplementary Table 13: Univariate analyses to identify factors predicting improvement in Nightmare Severity Index (NSI) Subscale 1 Nightmare Frequency (ΔNSI SS1).

*(NSI=Nightmare severity index ; ISI=Insomnia severity index ; ESS=Epworth sleepiness scale ; PSQI=Pittsburgh sleep quality index ; CES-D=Center for Epidemiologic Studies Depression Scale ; GAD-7= Generalized Anxiety Disorder-7; HAD=Hospital anxiety and depression scale ; QIDS-SR16=Quick inventory depressive symptoms-self report 16 items ; SUD=Substance use disorder ; MDE=Major depressive disorder ; TRD=Treatment resistant-depression ; MADRE=The Mannheim dream questionnaire ; PTSD=Post-Traumatic stress disorder ; GAD=Generalized Anxiety Disorder ; MADRS=Montgomery and Asberg Depression Rating Scale)*

|  | **Estimate (ß)** | **SE(ß)** | **Z** | **p** |
| --- | --- | --- | --- | --- |
| ***NSI SS1 improvement*** | | | | |
| **Age** | -0,0204 | 0,016 | -1,276 | 0,214 |
| **Gender** | 0,229 | 0,523 | 0,437 | 0,666 |
| ***Sleep parameters*** | | | | |
| **PSQI Sleep duration** | 0,0167 | 0,198 | 0,0843 | 0,934 |
| **PSQI Sleep subjective quality** | ^0,0547^ | 0,332 | ^0,165^ | 0,871 |
| **PSQI sleep latency** | -0,101 | 0,264 | -0,383 | 0,706 |
| **PSQI Daytime dysfunction** | -0,272 | 0,326 | -0,837 | 0,412 |
| **PSQI Sleep efficiency** | 0,169 | 0,207 | 0,817 | 0,422 |
| **PSQI Sleep disturbance** | 0,339 | 0,429 | 0,791 | 0,437 |
| **PSQI Sleep promoting medication** | -0,0645 | 0,192 | -0,336 | 0,740 |
| **PSQI** | 0,005 | 0,0789 | 0,0634 | 0,95 |
| **ESS** | 0,00342 | 0,0533 | 0,0642 | 0,949 |
| **Horne and Ostberg** | 0,0143 | 0,0315 | 0,455 | 0,653 |
| **NSI – Nightmares Severity** | -0,0691 | 0,0685 | -1,0083 | 0,323 |
| **NSI SS1 Frequency** | -0,2765 | 0,168 | -1,6494 | 0,112 |
| **NSI SS2 Emotional Impact** | 0,328 | 0,272 | 1,2 | 0,24 |
| **NSI SS3 Diurnal Impact** | -0,138 | 0,199 | -0,696 | 0,493 |
| **NSI SS4 Nocturnal Impact** | -0,25299 | 0,198 | -1,275 | 0,215 |
| **ISI Total score** | -0,00312 | 0,0524 | -0,0595 | 0,953 |
| **ISI1a - Severity of sleep latency** | -0,26 | 0,218 | -1,19 | 0,247 |
| **ISI1b - Difficulty staying asleep** | -0,108 | 0,21 | -0,513 | 0,613 |
| **ISI1c - Early morning awakening** | 0,0251 | 0,182 | 0,138 | 0,892 |
| **ISI2 - Satisfaction with current sleep** | 0,179 | 0,276 | 0,65 | 0,522 |
| **ISI3 - Disruption of daily functioning** | 0,183 | 0,241 | 0,763 | 0,423 |
| **ISI4 - Apparent difficulties** | 0,029 | 0,209 | 0,138 | 0,891 |
| **ISI5 - Concerns regarding sleep disturbances** | -0,0378 | 0,298 | -0,127 | 0,90 |
| ***Mood parameters*** | | | | |
| **MDE with anxiety** | 0,733 | 0,614 | 1,19 | 0,249 |
| **MDE with seasonal feature** | 0,291 | 0,640 | 0,455 | 0,656 |
| **MDE with TRD** | -0,889 | 0,806 | -1,1 | 0,284 |
| ***Severity of Depression and anxiety*** | | | | |
| **GAD-7** | 0,068 | 0,0387 | 1,76 | 0,091 |
| **HAD** | 0,0272 | 0,0612 | 0,445 | 0,661 |
| **HAD-D** | -0,0586 | 0,0625 | -0,939 | 0,358 |
| **HAD-A** | 0,0272 | 0,0612 | 0,445 | 0,661 |
| ***SUD*** | | | | |
| **SUD** | -0,361 | 0,747 | -0,483 | 0,634 |
| **Current tobacco smoking** | -0,383 | 0,583 | -0,658 | 0,518 |
| **Current alcohol use disorder** | -0,05 | 1,008 | -0,0496 | 0,961 |
| **Current cannabis use disorder** | 1 | 1,373 | 0,728 | 0,475 |
| ***Treatments*** | | | | |
| **Antidepressants** | 0,667 | 0,884 | 0,754 | 0,463 |
| **Anxiolytics** | 0,0571 | 0,679 | 0,0842 | 0,934 |
| **Mood stabilizers** | 0,462 | 0,711 | 0,65 | 0,527 |
| **Hypnotics** | 0,885 | 0,754 | 1,17 | 0,259 |
| **Antipsychotics** | 0,619 | 0,862 | 0,718 | 0,484 |
| ***Comorbidities*** | | | | |
| **PTSD** | -0,295 | 0,631 | -0,468 | 0,644 |
| **GAD** | -0,295 | 0,631 | -0,468 | 0,644 |

## Supplementary Table 14: Univariate analyses to identify factors predicting improvement in Nightmare Severity Index (NSI) Subscale 2 Nightmare Emotional Impact (ΔNSI SS2).

(*NSI=Nightmare severity index ; ISI=Insomnia severity index ; ESS=Epworth sleepiness scale ; PSQI=Pittsburgh sleep quality index ; CES-D=Center for Epidemiologic Studies Depression Scale ; GAD-7= Generalized Anxiety Disorder-7; HAD=Hospital anxiety and depression scale ; QIDS-SR16=Quick inventory depressive symptoms-self report 16 items ; SUD=Substance use disorder ; MDE=Major depressive disorder ; TRD=Treatment resistant-depression ; MADRE=The Mannheim dream questionnaire ; PTSD=Post-Traumatic stress disorder ; GAD=Generalized Anxiety Disorder ; MADRS=Montgomery and Asberg Depression Rating Scale)*

|  | **Estimate (ß)** | **SE(ß)** | **Z** | **p** |
| --- | --- | --- | --- | --- |
| ***NSI SS2 improvement*** | | | | |
| **Age** | -0,00663 | 0,0151 | -0,439 | 0,665 |
| **Gender** | -0,399 | 0,476 | -0,837 | 0,411 |
| ***Sleep parameters*** | | | | |
| **PSQI Sleep duration** | -0,123 | 0,169 | -0,725 | 0,476 |
| **PSQI Sleep subjective quality** | ^-0,271^ | 0,281 | ^-0,965^ | 0,345 |
| **PSQI sleep latency** | -0,184 | 0,225 | -0,817 | 0,423 |
| **PSQI Daytime dysfunction** | 0,138 | 0,288 | 0,478 | 0,638 |
| **PSQI Sleep efficiency** | -0,118 | 0,18 | -0,657 | 0,517 |
| **PSQI Sleep disturbance** | 0,228 | 0,372 | 0,612 | 0,546 |
| **PSQI Sleep promoting medication** | -0,172 | 0,131 | -1,07 | 0,298 |
| **PSQI** | -0,087 | 0,0656 | -1,3259 | 0,198 |
| **ESS** | -0,00998 | 0,0459 | -0,218 | 0,830 |
| **Horne and Ostberg** | -0,0309 | 0,0284 | -1,090 | 0,287 |
| **NSI – Nightmares Severity** | 0,114 | 0,0600 | 1,91 | 0,069 |
| **NSI SS1 Frequency** | 0,469 | 0,132 | 3,57 | **0,002** |
| **NSI SS2 Emotional Impact** | -0,183 | 0,255 | -0,716 | 0,481 |
| **NSI SS3 Diurnal Impact** | 0,163 | 0,181 | 0,898 | 0,378 |
| **NSI SS4 Nocturnal Impact** | 0,279 | 0,180 | 1,55 | 0,134 |
| **ISI Total score** | -0,0161 | 0,0451 | -0,357 | 0,724 |
| **ISI1a - Severity of sleep latency** | -0,200 | 0,190 | -1,05 | 0,303 |
| **ISI1b - Difficulty staying asleep** | -0,0285 | 0,182 | -0,157 | 0,877 |
| **ISI1c - Early morning awakening** | -0,190 | 0,152 | -1,25 | 0,223 |
| **ISI2 - Satisfaction with current sleep** | 0,0879 | 0,239 | 0,367 | 0,717 |
| **ISI3 - Disruption of daily functioning** | 0,113 | 0,209 | 0,544 | 0,592 |
| **ISI4 - Apparent difficulties** | 0,107 | 0,179 | 0,595 | 0,558 |
| **ISI5 - Concerns regarding sleep disturbances** | -0,0903 | 0,256 | -0,352 | 0,728 |
| ***Mood parameters*** | | | | |
| **MDE with anxiety** | 0,821 | 0,431 | 1,91 | 0,074 |
| **MDE with seasonal feature** | 0,271 | 0,532 | 0,510 | 0,616 |
| **MDE with TRD** | -1,722 | 0,553 | -3,11 | **0,006** |
| ***Severity of Depression and anxiety*** | | | | |
| **GAD-7** | 0,0328 | 0,0372 | 0,883 | 0,386 |
| **HAD** | -0,0304 | 0,0323 | -0,941 | 0,356 |
| **HAD-D** | -0,0538 | 0,0537 | -1,003 | 0,327 |
| **HAD-A** | -0,0286 | 0,0527 | -0,542 | 0,593 |
| ***SUD*** | | | | |
| **SUD** | 0,194 | 0,602 | 0,323 | 0,750 |
| **Current tobacco smoking** | -0,0667 | 0,496 | -0,134 | 0,894 |
| **Current alcohol use disorder** | -0,1000 | 0,809 | -0,124 | 0,903 |
| **Current cannabis use disorder** | 0,952 | 1,096 | 0,869 | 0,395 |
| ***Treatments*** | | | | |
| **Antidepressants** | -0,256 | 0,795 | -0,322 | 0,752 |
| **Anxiolytics** | -0,343 | 0,603 | -0,569 | 0,578 |
| **Mood stabilizers** | -0,897 | 0,784 | -1,14 | 0,272 |
| **Hypnotics** | -0,404 | 0,699 | -0,577 | 0,572 |
| **Antipsychotics** | -1,286 | 0,713 | -1,80 | 0,092 |
| ***Comorbidities*** | | | | |
| **PTSD** | -0,448 | 0,576 | -0,777 | 0,445 |
| **GAD** | 0,0476 | 0,583 | 0,0817 | 0,936 |

## Supplementary Table 15: Univariate analyses to identify factors predicting improvement in Nightmare Severity Index (NSI) Subscale 3 Nightmare Diurnal Impact (ΔNSI SS3).

*NSI=Nightmare severity index ; ISI=Insomnia severity index ; ESS=Epworth sleepiness scale ; PSQI=Pittsburgh sleep quality index ; CES-D=Center for Epidemiologic Studies Depression Scale ; GAD-7= Generalized Anxiety Disorder-7; HAD=Hospital anxiety and depression scale ; QIDS-SR16=Quick inventory depressive symptoms-self report 16 items ; SUD=Substance use disorder ; MDE=Major depressive disorder ; TRD=Treatment resistant-depression ; MADRE=The Mannheim dream questionnaire ; PTSD=Post-Traumatic stress disorder ; GAD=Generalized Anxiety Disorder ; MADRS=Montgomery and Asberg Depression Rating Scale)*

|  | **Estimate (ß)** | **SE(ß)** | **Z** | **p** |
| --- | --- | --- | --- | --- |
| ***NSI SS3 improvement*** | | | | |
| **Age** | -0,0262 | 0,0190 | -1,376 | 0,181 |
| **Gender** | 0,699 | 0,613 | 1,14 | 0,265 |
| ***Sleep parameters*** | | | | |
| **PSQI Sleep duration** | 0,197 | 0,234 | 0,843 | 0,408 |
| **PSQI Sleep subjective quality** | ^-0,354^ | 0,390 | ^-0,907^ | 0,374 |
| **PSQI sleep latency** | 0,209 | 0,314 | 0,665 | 0,513 |
| **PSQI Daytime dysfunction** | -0,2354 | 0,318 | -0,741 | 0,467 |
| **PSQI Sleep efficiency** | 0,191 | 0,249 | 0,770 | 0,449 |
| **PSQI Sleep disturbance** | -0,518 | 0,509 | -1,018 | 0,319 |
| **PSQI Sleep promoting medication** | -0,274 | 0,226 | -1,215 | 0,237 |
| **PSQI** | 0,00546 | 0,0944 | 0,0483 | 0,962 |
| **ESS** | 0,0174 | 0,0636 | 0,273 | 0,787 |
| **Horne and Ostberg** | -0,0471 | 0,0366 | -1,287 | 0,210 |
| **NSI – Nightmares Severity** | -0,112 | 0,0806 | -1,396 | 0,176 |
| **NSI SS1 Frequency** | -0,0408 | 0,212 | -0,193 | 0,849 |
| **NSI SS2 Emotional Impact** | 0,0448 | 0,336 | 0,133 | 0,895 |
| **NSI SS3 Diurnal Impact** | -0,525 | 0,215 | -2,44 | **0,022** |
| **NSI SS4 Nocturnal Impact** | -0,386 | 0,233 | -1,662 | 0,110 |
| **ISI Total score** | -0,0427 | 0,0621 | -0,6871 | 0,499 |
| **ISI1a - Severity of sleep latency** | 0,146 | 0,258 | 0,545 | 0,591 |
| **ISI1b - Difficulty staying asleep** | -0,30973 | 0,245 | -1,26553 | 0,218 |
| **ISI1c - Early morning awakening** | -0,3425 | 0,206 | -1,663 | 0,110 |
| **ISI2 - Satisfaction with current sleep** | -0,0330 | 0,333 | -0,0990 | 0,922 |
| **ISI3 - Disruption of daily functioning** | 0,181 | 0,289 | 0,625 | 0,538 |
| **ISI4 - Apparent difficulties** | 0,0797 | 0,250 | 0,319 | 0,753 |
| **ISI5 - Concerns regarding sleep disturbances** | -0,498 | 0,341 | -1,458 | 0,158 |
| ***Mood parameters*** | | | | |
| **MDE with anxiety** | 1,1 | 0,619 | 1,78 | 0,094 |
| **MDE with seasonal feature** | -0,243 | 0,762 | -0,319 | 0,754 |
| **MDE with TRD** | 0,2 | 0,927 | 0,216 | 0,832 |
| ***Severity of Depression and anxiety*** | | | | |
| **GAD-7** | 0,0757 | 0,0467 | 1,62 | 0,118 |
| **HAD** | 0,0793 | 0,0426 | 1,86 | 0,075 |
| **HAD-D** | 0,0725 | 0,0746 | 0,972 | 0,341 |
| **HAD-A** | 0,138 | 0,0677 | 2,04 | 0,053 |
| ***SUD*** | | | | |
| **SUD** | -0,722 | 0,695 | -1,039 | 0,311 |
| **Current tobacco smoking** | -0,158 | 0,631 | -0,251 | 0,804 |
| **Current alcohol use disorder** | -1,200 | 0,919 | -1,31 | 0,206 |
| **Current cannabis use disorder** | 0,429 | 1,318 | 0,325 | 0,748 |
| ***Treatments*** | | | | |
| **Antidepressants** | -1,128 | 0,638 | -1,77 | 0,099 |
| **Anxiolytics** | 0,0143 | 0,533 | 0,0268 | 0,979 |
| **Mood stabilizers** | -0,359 | 0,673 | -0,534 | 0,602 |
| **Hypnotics** | 0,0577 | 0,618 | 0,0934 | 0,927 |
| **Antipsychotics** | -0,452 | 0,678 | -0,667 | 0,515 |
| ***Comorbidities*** | | | | |
| **PTSD** | 0,0667 | 0,759 | 0,0878 | 0,931 |
| **GAD** | 0,562 | 0,751 | 0,759 | 0,461 |

## Supplementary Table 16: Univariate analyses to identify factors predicting improvement in Nightmare Severity Index (NSI) Subscale 4 Nightmare Nocturnal Impact (ΔNSI SS4).

*NSI=Nightmare severity index ; ISI=Insomnia severity index ; ESS=Epworth sleepiness scale ; PSQI=Pittsburgh sleep quality index ; CES-D=Center for Epidemiologic Studies Depression Scale ; GAD-7= Generalized Anxiety Disorder-7; HAD=Hospital anxiety and depression scale ; QIDS-SR16=Quick inventory depressive symptoms-self report 16 items ; SUD=Substance use disorder ; MDE=Major depressive disorder ; TRD=Treatment resistant-depression ; MADRE=The Mannheim dream questionnaire ; PTSD=Post-Traumatic stress disorder ; GAD=Generalized Anxiety Disorder ; MADRS=Montgomery and Asberg Depression Rating Scale)*

|  | **Estimate (ß)** | **SE(ß)** | **Z** | **p** |
| --- | --- | --- | --- | --- |
| ***NSI SS4 improvement*** | | | | |
| **Age** | -3,22e^-4^ | 0,0149 | -0,0216 | 0,983 |
| **Gender** | -0,222 | 0,473 | -0,470 | 0,643 |
| ***Sleep parameters*** | | | | |
| **PSQI Sleep duration** | 0,110 | 0,175 | 0,625 | 0,538 |
| **PSQI Sleep subjective quality** | ^-0,336^ | 0,288 | ^-1,168^ | 0,255 |
| **PSQI sleep latency** | 0,272 | 0,229 | 1,18 | 0,248 |
| **PSQI Daytime dysfunction** | -0,683 | 0,234 | -2,913 | **0,008** |
| **PSQI Sleep efficiency** | 0,0126 | 0,188 | 0,0669 | 0,947 |
| **PSQI Sleep disturbance** | -0,665 | 0,362 | -1,838 | 0,079 |
| **PSQI Sleep promoting medication** | 0,0806 | 0,160 | 0,503 | 0,620 |
| **PSQI** | 0,00132 | 0,0704 | 0,0188 | 0,985 |
| **ESS** | -0,0887 | 0,0437 | -2,0285 | 0,054 |
| **Horne and Ostberg** | -0,0169 | 0,0284 | -0,5957 | 0,557 |
| **NSI – Nightmares Severity** | -0,0658 | 0,0618 | -1,0654 | 0,297 |
| **NSI SS1 Frequency** | -0,0519 | 0,160 | -0,325 | 0,748 |
| **NSI SS2 Emotional Impact** | 0,269 | 0,248 | 1,08 | 0,289 |
| **NSI SS3 Diurnal Impact** | -0,202 | 0,177 | -1,144 | 0,264 |
| **NSI SS4 Nocturnal Impact** | -0,428 | 0,163 | -2,62 | **0,015** |
| **ISI Total score** | -0,00799 | 0,0467 | -0,0171 | 0,866 |
| **ISI1a - Severity of sleep latency** | 0,0395 | 0,200 | 0,197 | 0,846 |
| **ISI1b - Difficulty staying asleep** | -0,0550 | 0,188 | -0,292 | 0,773 |
| **ISI1c - Early morning awakening** | -0,190 | 0,157 | -1,21 | 0,239 |
| **ISI2 - Satisfaction with current sleep** | -0,141 | 0,247 | -0,572 | 0,573 |
| **ISI3 - Disruption of daily functioning** | 0,113 | 0,216 | 0,526 | 0,604 |
| **ISI4 - Apparent difficulties** | 0,00311 | 0,187 | 0,0166 | 0,987 |
| **ISI5 - Concerns regarding sleep disturbances** | 0,277 | 0,260 | 1,07 | 0,296 |
| ***Mood parameters*** | | | | |
| **MDE with anxiety** | 0,956 | 0,454 | 2,10 | 0,051 |
| **MDE with seasonal feature** | 0,343 | 0,572 | 0,599 | 0,557 |
| **MDE with TRD** | 0,533 | 0,759 | 0,702 | 0,493 |
| ***Severity of Depression and anxiety*** | | | | |
| **GAD-7** | 0,0424 | 0,0361 | 1,18 | 0,251 |
| **HAD** | 0,0316 | 0,0334 | 0,946 | 0,354 |
| **HAD-D** | 0,0585 | 0,0554 | 1,06 | 0,302 |
| **HAD-A** | 0,0272 | 0,0545 | 0,499 | 0,622 |
| ***SUD*** | | | | |
| **SUD** | 0,194 | 0,627 | 0,310 | 0,760 |
| **Current tobacco smoking** | -0,258 | 0,529 | -0,489 | 0,630 |
| **Current alcohol use disorder** | -0,100 | 0,842 | -0,119 | 0,907 |
| **Current cannabis use disorder** | 0,952 | 1,143 | 0,833 | 0,415 |
| ***Treatments*** | | | | |
| **Antidepressants** | 0,333 | 0,815 | 0,409 | 0,689 |
| **Anxiolytics** | 0,829 | 0,571 | 1,45 | 0,167 |
| **Mood stabilizers** | -0,179 | 0,733 | -0,245 | 0,810 |
| **Hypnotics** | 0,0769 | 0,707 | 0,109 | 0,915 |
| **Antipsychotics** | 0,0714 | 0,787 | 0,0908 | 0,929 |
| ***Comorbidities*** | | | | |
| **PTSD** | -0,838 | 0,547 | -1,53 | 0,139 |
| **GAD** | -0,0952 | 0,573 | -0,166 | 0,869 |

## Supplementary Table 17: Logistic regression to identify factors predicting Nightmare Severity Index (NSI) 33% Response.

*(NSI=Nightmare severity index ; ISI=Insomnia severity index ; ESS=Epworth sleepiness scale ; PSQI=Pittsburgh sleep quality index ; CES-D=Center for Epidemiologic Studies Depression Scale ; GAD-7= Generalized Anxiety Disorder-7; HAD=Hospital anxiety and depression scale ; QIDS-SR16=Quick inventory depressive symptoms-self report 16 items ; SUD=Substance use disorder ; MDE=Major depressive disorder ; TRD=Treatment resistant-depression ; MADRE=The Mannheim dream questionnaire ; PTSD=Post-Traumatic stress disorder ; GAD=Generalized Anxiety Disorder ; MADRS=Montgomery and Asberg Depression Rating Scale)*

|  | **Estimate (ß)** | **SE(ß)** | **Z** | **p** |
| --- | --- | --- | --- | --- |
| ***NSI 33% Response*** | | | | |
| **Age** | 0,0131 | 0,0271 | 0,484 | 0,628 |
| **Gender** | -0,652 | 0,856 | -0,762 | 0,446 |
| ***Sleep parameters*** | | | | |
| **PSQI Sleep duration** | -0,363 | 0,348 | -1,043 | 0,297 |
| **PSQI Sleep subjective quality** | ^0,570^ | 0,617 | ^0,923^ | 0,356 |
| **PSQI sleep latency** | 0,0917 | 0,439 | 0,209 | 0,834 |
| **PSQI Daytime dysfunction** | 0,538 | 0,558 | 0,964 | 0,335 |
| **PSQI Sleep efficiency** | -0,673 | 0,393 | -1,709 | 0,087 |
| **PSQI Sleep disturbance** | 0,164 | 0,718 | 0,229 | 0,819 |
| **PSQI Sleep promoting medication** | 0,0979 | 0,315 | 0,311 | 0,756 |
| **PSQI** | -0,0531 | 0,131 | -0,4046 | 0,686 |
| **ESS** | 0,0719 | 0,0890 | 0,808 | 0,419 |
| **Horne and Ostberg** | 0,0978 | 0,0598 | 1,63 | 0,102 |
| **NSI – Nightmares Severity** | -0,170 | 0,124 | -1,37 | 0,169 |
| **NSI SS1 Frequency** | -0,547 | 0,326 | -1,68 | 0,093 |
| **NSI SS2 Emotional Impact** | -0,227 | 0,458 | -0,496 | 0,620 |
| **NSI SS3 Diurnal Impact** | -0,238 | 0,330 | -0,719 | 0,472 |
| **NSI SS4 Nocturnal Impact** | -0,330 | 0,339 | -0,974 | 0,330 |
| **ISI Total score** | -0,0102 | 0,0870 | -0,117 | 0,907 |
| **ISI1a - Severity of sleep latency** | 0,0769 | 0,370 | 0,207 | 0,836 |
| **ISI1b - Difficulty staying asleep** | -0,198 | 0,347 | -0,569 | 0,570 |
| **ISI1c - Early morning awakening** | 0,0216 | 0,300 | 0,0720 | 0,943 |
| **ISI2 - Satisfaction with current sleep** | 0,315 | 0,511 | 0,616 | 0,538 |
| **ISI3 - Disruption of daily functioning** | -0,335 | 0,406 | -0,826 | 0,409 |
| **ISI4 - Apparent difficulties** | -0,00476 | 0,345 | -0,0138 | 0,989 |
| **ISI5 - Concerns regarding sleep disturbances** | 0,0580 | 0,492 | 0,118 | 0,906 |
| ***Mood parameters*** | | | | |
| **MDE with anxiety** | -1,974 | 1,249 | -1,580 | 0,114 |
| **MDE with seasonal feature** | -0,470 | 1,265 | -0,372 | 0,710 |
| **MDE with TRD** | 0,318 | 1,357 | 0,235 | 0,814 |
| ***Severity of Depression and anxiety*** | | | | |
| **GAD-7** | -0,147 | 0,0789 | -1,87 | 0,062 |
| **HAD** | -0,00615 | 0,0627 | -0,0981 | 0,922 |
| **HAD-D** | 0,0526 | 0,104 | 0,505 | 0,614 |
| **HAD-A** | -0,0649 | 0,101 | -0,6441 | 0,519 |
| ***SUD*** | | | | |
| **SUD** | 1,25 | 1,150 | 1,09 | 0,276 |
| **Current tobacco smoking** | -0,405 | 0,983 | -0,412 | 0,680 |
| **Current alcohol use disorder** | 1,10 | 1,506 | 0,730 | 0,466 |
| **Current cannabis use disorder** | -15,650 | 2399,545 | -0,00652 | 0,995 |
| ***Treatments*** | | | | |
| **Antidepressants** | 0,223 | 1,35 | 0,165 | 0,869 |
| **Anxiolytics** | -0,511 | 1,057 | -0,483 | 0,629 |
| **Mood stabilizers** | 1,504 | 1,364 | 1,10 | 0,270 |
| **Hypnotics** | -0,629 | 1,288 | -0,488 | 0,625 |
| **Antipsychotics** | -0,105 | 1,346 | -0,0783 | 0,938 |
| ***Comorbidities*** | | | | |
| **PTSD** | -0,0870 | 0,870 | -0,1000 | 0,920 |
| **GAD** | 0,288 | 1,024 | 0,281 | 0,7**7**9 |

## Supplementary Table 18: Logistic regression to identify factors predicting Nightmare Severity Index (NSI) 25% Response.

(*NSI=Nightmare severity index ; ISI=Insomnia severity index ; ESS=Epworth sleepiness scale ; PSQI=Pittsburgh sleep quality index ; CES-D=Center for Epidemiologic Studies Depression Scale ; GAD-7= Generalized Anxiety Disorder-7; HAD=Hospital anxiety and depression scale ; QIDS-SR16=Quick inventory depressive symptoms-self report 16 items ; SUD=Substance use disorder ; MDE=Major depressive disorder ; TRD=Treatment resistant-depression ; MADRE=The Mannheim dream questionnaire ; PTSD=Post-Traumatic stress disorder ; GAD=Generalized Anxiety Disorder ; MADRS=Montgomery and Asberg Depression Rating Scale)*

|  | **Estimate (ß)** | **SE(ß)** | **Z** | **p** |
| --- | --- | --- | --- | --- |
| ***NSI 25% Response*** | | | | |
| **Age** | 0,0314 | 0,0270 | 1,16 | 0,245 |
| **Gender** | -0,580 | 0,832 | -0,697 | 0,486 |
| ***Sleep parameters*** | | | | |
| **PSQI Sleep duration** | -0,102 | 0,309 | -0,331 | 0,740 |
| **PSQI Sleep subjective quality** | ^0,0636^ | 0,516 | ^0,123^ | 0,902 |
| **PSQI sleep latency** | 0,372 | 0,425 | 0,876 | 0,381 |
| **PSQI Daytime dysfunction** | 0,151 | 0,499 | 0,302 | 0,762 |
| **PSQI Sleep efficiency** | -0,293 | 0,333 | -0,879 | 0,379 |
| **PSQI Sleep disturbance** | -0,255 | 0,678 | -0,376 | 0,707 |
| **PSQI Sleep promoting medication** | 0,432 | 0,315 | 1,37 | 0,171 |
| **PSQI** | 0,0596 | 0,123 | 0,482 | 0,630 |
| **ESS** | -0,0382 | 0,0836 | -0,457 | 0,648 |
| **Horne and Ostberg** | 0,0573 | 0,0531 | 1,08 | 0,280 |
| **NSI – Nightmares Severity** | -0,0252 | 0,110 | -0,229 | 0,819 |
| **NSI SS1 Frequency** | -0,168 | 0,282 | -0,596 | 0,551 |
| **NSI SS2 Emotional Impact** | 0,0451 | 0,443 | 0,102 | 0,919 |
| **NSI SS3 Diurnal Impact** | 0,00767 | 0,316 | 0,0243 | 0,981 |
| **NSI SS4 Nocturnal Impact** | -0,0240 | 0,323 | -0,0745 | 0,941 |
| **ISI Total score** | 0,0124 | 0,0811 | 0,153 | 0,879 |
| **ISI1a - Severity of sleep latency** | 0,447 | 0,375 | 1,19 | 0,232 |
| **ISI1b - Difficulty staying asleep** | 0,0129 | 0,328 | 0,0393 | 0,969 |
| **ISI1c - Early morning awakening** | 0,333 | 0,294 | 1,13 | 0,258 |
| **ISI2 - Satisfaction with current sleep** | -0,357 | 0,451 | -0,792 | 0,429 |
| **ISI3 - Disruption of daily functioning** | -0,493 | 0,397 | -1,241 | 0,215 |
| **ISI4 - Apparent difficulties** | -0,287 | 0,333 | -0,861 | 0,390 |
| **ISI5 - Concerns regarding sleep disturbances** | 0,458 | 0,484 | 0,947 | 0,344 |
| ***Mood parameters*** | | | | |
| **MDE with anxiety** | -2,079 | 1,061 | -1,961 | 0,050 |
| **MDE with seasonal feature** | -0,118 | 1,061 | -0,111 | 0,912 |
| **MDE with TRD** | 1,099 | 1,333 | 0,824 | 0,410 |
| ***Severity of Depression and anxiety*** | | | | |
| **GAD-7** | -0,143 | 0,0758 | -1,89 | 0,058 |
| **HAD** | 0,0193 | 0,0597 | 0,323 | 0,746 |
| **HAD-D** | 0,103 | 0,104 | 0,991 | 0,322 |
| **HAD-A** | -0,0435 | 0,0955 | -0,456 | 0,649 |
| ***SUD*** | | | | |
| **SUD** | 0,452 | 1,111 | 0,407 | 0,684 |
| **Current tobacco smoking** | -0,405 | 0,983 | -0,412 | 0,680 |
| **Current alcohol use disorder** | 0,405 | 1,486 | 0,273 | 0,785 |
| **Current cannabis use disorder** | -16,278 | 2399,545 | -0,00678 | 0,995 |
| ***Treatments*** | | | | |
| **Antidepressants** | 0,539 | 1,35 | 0,401 | 0,689 |
| **Anxiolytics** | 0,693 | 1 | 0,693 | 0,488 |
| **Mood stabilizers** | 0,847 | 1,345 | 0,630 | 0,529 |
| **Hypnotics** | 0,154 | 1,144 | 0,135 | 0,893 |
| **Antipsychotics** | 0,981 | 1,339 | 0,733 | 0,464 |
| ***Comorbidities*** | | | | |
| **PTSD** | 0,693 | 1,014 | 0,684 | 0,494 |
| **GAD** | -0,3102 | 1,012 | -0,306 | 0,759 |
